# Creatine Kinase Equilibration and ΔG_ATP_ over an Extended Range of Physiological Conditions: Implications for Cellular Energetics, Signaling, and Muscle Performance

**DOI:** 10.3390/ijms241713244

**Published:** 2023-08-26

**Authors:** Robert Woodbury Wiseman, Caleb Micah Brown, Thomas Wesley Beck, Jeffrey John Brault, Tyler Robert Reinoso, Yun Shi, Prescott Bryant Chase

**Affiliations:** 1 Departments of Physiology and Radiology, Michigan State University, East Lansing, MI 48824, USA; rwiseman@msu.edu; 2 Department of Biochemistry, University of Washington, Seattle, WA 98195, USA; 3 Department of Radiology, University of Washington, Seattle, WA 98195, USA; 4 Department of Physiology, Michigan State University, East Lansing, MI 48824, USA; jebrault@iu.edu; 5 Department of Biological Science, Florida State University, Tallahassee, FL 32306, USA

**Keywords:** skeletal muscle, actomyosin ATPase, ADP, temperature, cytoplasmic magnesium ion concentration, cytoplasmic pH, inorganic phosphate, Pi, isometric force generation

## Abstract

In this report, we establish a straightforward method for estimating the equilibrium constant for the creatine kinase reaction (CK K_eq_″) over wide but physiologically and experimentally relevant ranges of pH, Mg^2+^ and temperature. Our empirical formula for CK K_eq_″ is based on experimental measurements. It can be used to estimate [ADP] when [ADP] is below the resolution of experimental measurements, a typical situation because [ADP] is on the order of micromolar concentrations in living cells and may be much lower in many in vitro experiments. Accurate prediction of [ADP] is essential for in vivo studies of cellular energetics and metabolism and for in vitro studies of ATP-dependent enzyme function under near-physiological conditions. With [ADP], we were able to obtain improved estimates of ΔG_ATP_, necessitating the reinvestigation of previously reported ADP- and ΔG_ATP_-dependent processes. Application to actomyosin force generation in muscle provides support for the hypothesis that, when [Pi] varies and pH is not altered, the maximum Ca^2+^-activated isometric force depends on ΔG_ATP_ in both living and permeabilized muscle preparations. Further analysis of the pH studies introduces a novel hypothesis around the role of submicromolar ADP in force generation.

## 1. Introduction

Energy homeostasis is a fundamental property of all cells that is achieved through matching ATP synthesis with its use. ATP free energy (ΔG_ATP_) homeostasis in myocytes is critical because ATP hydrolysis provides the driving force for both actin–myosin interactions and Ca^2+^ transport, cellular functions that vary markedly between rest and activation in muscle [1]. Dysregulation of ΔG_ATP_ can have negative consequences for health [2,3,4,5] and longevity [6,7], and is postulated to play a central role in muscle growth and diseases such as cardiomyopathies, heart failure, obesity, and type 2 diabetes [8,9,10,11,12,13,14,15,16,17,18,19,20,21,22].

Understanding the energetic relationships between ΔG_ATP_ and the enzymes that utilize ATP, as well as the metabolic pathways that generate ATP, requires precise knowledge of the free energy available under both in vivo and experimental conditions [23,24]. The essentially irreversible hydrolysis reaction for ATP takes the balanced form [24,25,26,27].

ATP^4−^ → ADP^3−^ + HPO_4_^2−^ + H^+^ (1)

Because the major cellular ATPases utilize adenine nucleotides complexed with Mg^2+^ and produce inorganic phosphate (Pi) and a non-stoichiometrically generated proton (H^+^), the ATP hydrolysis reaction in cells can be rewritten more generally as

MgATP^2−^ → MgADP^−^ + Pi + αH^+^ (2)

A simple definition of ΔG_ATP_ that implicitly incorporates the nuances of Equations (1) and (2) can be written as

(3) $$\Delta G_{ATP}=\Delta G_{ATP}^{0}+RT\ln\left(\frac{\left[ADP\right]\left[Pi\right]}{\left[ATP\right]}\right)$$

where ΔG^0^_ATP_ is the free energy of ATP hydrolysis under standard conditions of temperature, pressure, and substrate/product concentrations, T is the temperature in °K, and R is the gas constant. In a healthy cell, ΔG_ATP_ is on the order of 100 pN·nm per molecule of ATP, which provides an upper limit to the thermodynamic efficiency of work performed by a cellular ATPase [28]. The exact values of ΔG_ATP_ and ΔG^0^_ATP_, however, can vary significantly in the steady-state, both physiologically and experimentally, as implied by Equations (1)–(3). ΔG_ATP_ not only varies with changes in [ATP], [ADP], and [Pi] (Equation (3)); both ΔG_ATP_ and ΔG^0^_ATP_ are influenced by changes in pH, [Mg^2+^], and other physicochemical parameters [29]. ΔG_ATP_ and ΔG^0^_ATP_ can vary dramatically as these parameters (especially Mg^2+^) are altered, mainly due to formation of non-covalent complexes and the associated binding enthalpies of ions with adenine nucleotides and Pi [23,24]. Of the parameters needed to estimate ΔG_ATP_, measurement of cytoplasmic ADP is particularly challenging. The cytoplasmic concentration of free ADP in healthy cells is typically below the limit of detection for direct measurement in vivo (e.g., by ^31^P-NMR) [1,30,31,32,33]. In addition, it is only a small fraction of the total ADP in a cell, which includes protein-bound ADP (e.g., ADP bound to actin in the living cell) that is released during tissue processing to extract ADP for analysis. Further, the collection and extraction process may artifactually increase ADP due to hydrolysis of a fraction of the much greater amounts of ATP [34].

In living cells and in many experiments with skinned muscle fibers [35,36], [ATP], [ADP], and ΔG_ATP_ are buffered by the creatine kinase (CK) or Lohmann reaction. CK catalyzes the reversible transfer of phosphate between phosphocreatine (PCr) and MgADP to resynthesize MgATP:

PCr^2−^ + MgADP^−^ + βH^+^ ↔ Cr + MgATP^2^ (4)

The proton stoichiometric coefficient β (Equation (4)) is analogous to the coefficient α for ATP hydrolysis (Equation (2)). Because of the large amount of CK in striated muscle and its high activity, the reaction catalyzed by this enzyme is likely to be at or near equilibrium under most conditions. Thus, ΔG_ATP_ in vivo is intimately linked to the CK reaction, and an assumption of near equilibrium can be used to first calculate free cytosolic ADP en route to estimating ΔG_ATP_. The equilibrium constant K_eq_ for the reaction catalyzed by CK (Equation (4)) is

(5) $$K_{eq}=\frac{\left[Cr\right]\left[{MgATP}^{2-}\right]}{\left[{PCr}^{2-}\right]\left[{MgADP}^{-}\right]{\left[H^{+}\right]}^{\beta }}$$

Equation (5) is often rewritten in simplified form as an apparent equilibrium constant (K_eq_′) [29,37]:

(6) $$K_{eq}^{\prime }\left[pH,pMg,T\right]=\frac{\sum \left[Cr\right]\sum \left[ATP\right]}{\sum \left[PCr\right]\sum \left[ADP\right]}$$

Equation (6) is compatible with most analytical measurements at a given pH, because there is no attempt to distinguish among the various ionic species such as those included in Equation (5). Each sum in Equation (6) includes all of the relevant ionic species, including minor species, for example, for ATP:

Σ[ATP] = [ATP^4−^] + [HATP^3−^] + [H_2_ATP^2−^] + [H_3_ATP^−^] + [H_4_ATP] + [MgATP^2−^] + [MgHATP^−^] + [Mg_2_ATP] + [CaATP^2−^] + [CaHATP^−^] + [NaATP^3−^] + [KATP^3−^]) (7)

When K_eq_′ has been determined, Equation (6) can be of utility in metabolic studies for estimating the cytoplasmic concentration of free ADP and, in combination with Equation (3), ΔG_ATP_. While K_eq_′ (Equation (6)) is proportional to K_eq_ (Equation (5)), the constant of proportionality varies with pH, temperature, free Mg^2+^ (reported as pMg = −log [Mg^2+^], where [Mg^2+^] is in molar units), etc., which places a severe limitation on how broadly any estimate of K_eq_′ [pH, pMg, T] can be applied. Many of the same parameters that affect ΔG_ATP_ and ΔG^0^_ATP_ (Equation (3)), including [Mg^2+^] and pH, along with the temperature and ionic strength (Γ/2), affect K_eq_′ (Equation (6)). A comprehensive empirical approach to determine [ADP] for calculating ΔG_ATP_ must account for variations of these parameters in living muscle as well as for in vitro studies in order to accurately assess both free energy changes and their physiological consequences.

Central to detailed models of the actomyosin crossbridge cycle is the thermodynamic constraint that ΔG_ATP_ is a primary determinant of steady-state isometric force [38,39,40,41]. Furthermore, a quasilinear relation has been identified between ΔG_ATP_ and ATP hydrolysis flux when constant pH is maintained [42,43]. Elevated Pi reduces the maximum isometric force in skinned muscle fibers [35,44,45,46,47,48]. The inverse correlation between maximum isometric force and Pi observed in skinned fibers has been confirmed in isolated intact slow-twitch muscle from mice, where lowering Pi resulted in an increase in maximum isometric force [49]. Because ΔG_ATP_ varies inversely with Pi (Equation (3)), the observed relationship that the isometric force varies with changes in Pi provides strong support for an energetic constraint on the molecular mechanism of force generation by actomyosin in both skinned fibers and in isolated intact muscles. In accordance with this concept, Karatzaferi et al. [45] reported that the maximum isometric force varies with the change in free energy when [Pi] is varied over several orders of magnitude, leading to the idea that free energy determines isometric force through its influence on actomyosin bond strength. The generality of physiological and experimental circumstances in which it can be directly applied to understand muscle function has not been fully examined. While one could consider varying ΔG_ATP_ through changes in [ATP] and/or [ADP] according to the definition of ΔG_ATP_ (Equation (3)), [ATP] and [ADP] are more challenging to vary in a controlled and independent manner [36,50,51,52]. The multiple influences of pH (Equations (2) and (4) plus the involvement of [H^+^] in ion-binding equilibria) further contribute to the challenge of obtaining accurate estimates of ΔG_ATP_, which has prevented rigorous empirical tests of whether cellular ATP-driven processes, molecular motors in particular, can vary either their coupling to or work performed by ΔG_ATP_, particularly in light of large physiological fluctuations in ΔG_ATP_ [35,42,43,44,45,47].

In view of the central role of the CK reaction for determining ΔG_ATP_ in many cell types, an important biochemical goal of this study was, first, a quantitative measurement of K_eq_′ for the CK reaction (Equation (6)) across a broad range of physiological and experimentally relevant pH, [Mg^2+^], and temperatures while holding Γ/2 constant. With these results, we could use readily determined concentrations of ATP, PCr, and Cr to estimate [ADP] for any combination of pH, [Mg^2+^], and temperature within the ranges examined. This empirical analysis produced a comprehensive quantitative adjustment of the equilibrium constant across key differences in physiological parameters, permitting direct comparisons of ADP and ΔG_ATP_ among disparate studies.

The relationship between ΔG_ATP_ and mechanical output (e.g., isometric force) could then be examined quantitatively using results from skinned fibers and isolated muscles. We present both in vitro and in vivo confirmations of the previously reported reciprocal relationship between isometric force and Pi demonstrated in both skinned [35,44,45,46,47,48] and intact muscles [49] as well as the corresponding relationship between isometric force and ΔG_ATP_ when Pi is varied [45]. While the relationship between ΔG_ATP_ and pH is complex, it can be readily predicted using the results of this study. We show here that the relationship between isometric force and ΔG_ATP_ when pH is varied is different from that obtained with Pi when ΔG_ATP_ is modulated by changing pH in either chemically skinned fibers [35] or intact muscle preparations [53]. These results suggest that the effects of Pi on actomyosin are directly modulated through free energy changes, while the effects of pH on force may be primarily due to other factors, possibly including [ADP]. The methods described here are generally applicable to studies of cellular energetics and mathematical modeling of metabolic flux in striated muscles, including myocardial bioenergetics [54].

## 2. Results

### 2.1. ^31^P-NMR Analysis of Solutions

**Figure 1:**
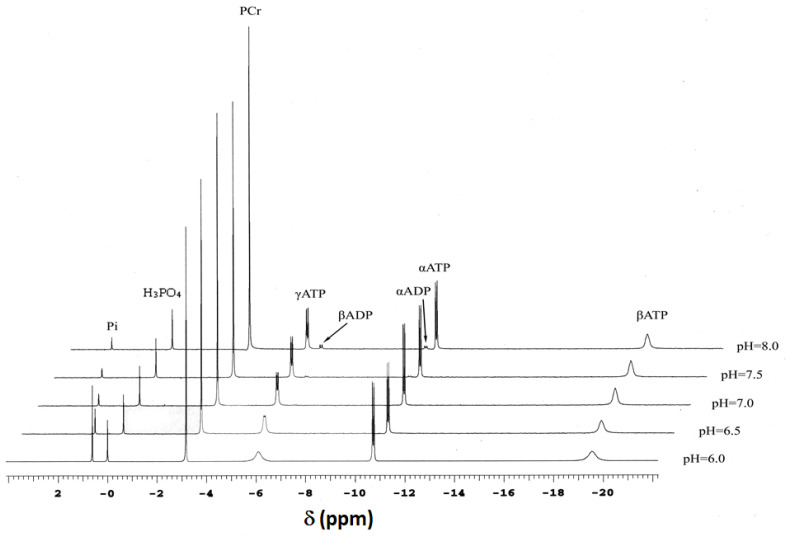
^31^P-NMR spectroscopy of model solutions containing inorganic phosphate (Pi), phosphocreatine (PCr), ATP (left-to-right, the peaks correspond to the γ, α, and β phosphate resonances), ADP (only visible at pH 7.5 and 8.0; left-to-right, the peaks correspond to the β and α phosphate resonances) in the presence of CK and with 50 mM added Cr at pMg 3.0, 30 °C, and pH 6.0, 6.5, 7.0, 7.5, or 8.0 (bottom-to-top stacked plot). Details of solution composition were as described in Section 4.1. An external standard (H_3_PO_4_, chemical shift δ = 0) was placed in a small capillary and centered in the coil’s sensitive volume. Spectra were acquired on a Varian 600 MHz spectrometer at the phosphorus frequency (242 MHz) using an 8000 Hz sweep width. Data are the sum of 1024 transients collected with a 1.0 s recycle delay and a π/2 pulse width and 1024 complex points and zero-filled once to a total of 2048 data points and exponentially filtered prior to the Fourier transform. Note that Pi chemical shift (δ_Pi_) moves from right to left along the δ-axis with increasing pH (bottom-to-top), corresponding to deprotonation of H_2_PO_4_^−^ with pK_a_ around neutral pH. Additionally, note that the γATP and βATP peaks become sharper with increasing pH (bottom-to-top) and that peak splitting is evident under most conditions for the γATP and αATP peaks, as well as for the βADP and αADP peaks where they are detectable.

Solutions that mimic the intracellular environment (Section 4.1) were first analyzed by ^31^P-NMR. Figure 1 shows a representative series of ^31^P-NMR spectra obtained over the entire pH range (five discrete pH values: pH 6.0, 6.5, 7.0, 7.5, and 8.0), pMg 3.0, 30 °C, and 50 mM Cr added. ^31^P-NMR was used to validate significant aspects of solution composition and to demonstrate that equilibrium was achieved following addition of CK and prior to termination of the reaction for further analyses.

**Figure 2:**
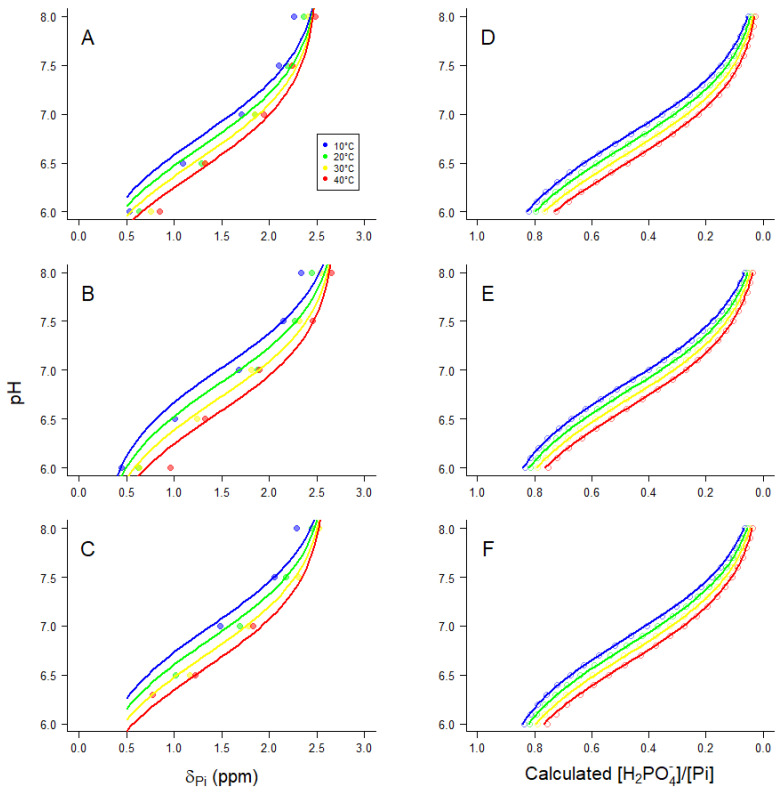
(**A**–**C**) Experimentally determined ^31^P-NMR chemical shift of Pi (δ_Pi_) titrated between pH 6–8 and (**D**–**F**) calculated pH-dependence of [H_2_PO_4_^−^]/Σ[Pi]. (**A**,**D**) pMg 2; (**B**,**E**) pMg 3; (**C**,**F**) pMg 4. In all panels, blue is 10 °C, green is 20 °C, yellow is 30 °C, and red is 40 °C. In panels (**A**–**C**), all data in each panel (single pMg) were simultaneously fitted to Equation (8) by nonlinear least squares regression, while in panels (**D**–**F**) all values in each panel (single pMg) were simultaneously fitted to Equation (9) by nonlinear least squares regression; regression parameter estimates are provided in Table 1. Note the qualitative correspondence between the left and right panels.

^31^P-NMR spectra obtained at equilibrium (Figure 1) allowed determination of a pK_a_ for H^+^ binding by Pi within the pH range 6–8 along with how that pK_a_ is affected by temperature and pMg (Figure 2). Such information is useful for calibration of pH_i_ in living tissue by evaluating the chemical shift of Pi relative to PCr (note that the chemical shift of PCr relative to an external standard of H_3_PO_4_ is −2.54 ppm, and is essentially constant over the physiological pH range). These relationships were quantified as adapted from Kost [55]:

(8) $$pH={pK}_{a}+\frac{d{pK}_{a}}{dt}\left(T-20\right)+log\left[\frac{∆-0.003579T-\delta_{Pi}}{\delta_{Pi}-∆+0.001888T-2.345}\right]$$

where δ_Pi_ is the ^31^P-NMR chemical shift difference between Pi at a given pH and the external standard (see spectra in Figure 1). The temperature dependencies of the extreme acid chemical shift δ_A_(T), the extreme basic chemical shift δ_B_(T), and the difference between them were consistent with, and were assumed to be the same as those described by Kost [55]. The variable Δ in Equation (8) was necessary to allow for a small offset in chemical shift between the current dataset and the values presented in Kost [55]. Figure 2A–C shows ^31^P-NMR chemical shift titrations for Pi (δ_Pi_) at 10 °C (blue), 20 °C (green), 30 °C (yellow), and 40 °C (red) at pMg 2.0 (Figure 2A), pMg 3.0 (Figure 2B), and pMg 4.0 (Figure 2C). All data at each pMg were simultaneously fitted to Equation (8); the resulting fits are shown in Figure 2A–C and the parameter estimates are provided in Table 1.

The coefficients from the chemical shift data (Figure 2A–C and Table 1) were corroborated with calculations from ion binding equilibria that were used for calculating the solution composition (Section 4.1), fitted to the following relationship:

(9) $$\frac{\left[H_{2}PO_{4}^{-}\right]}{\sum \left[Pi\right]}=\frac{A}{1+10^{\left(pH-\left({pK}_{a}+\frac{{dpK}_{a}}{dT}\left(T-20\right)\right)\right)}}$$

where Σ[Pi] is the sum over all relevant ionic forms of Pi, and has the same form as Equation (7) describing Σ[ATP] (Section 4.1):

Σ[Pi] = [PO_4_^3−^] + [HPO_4_^2−^] + [H_2_PO_4_^−^] + [H_3_PO_4_] + [MgHPO_4_] + [CaHPO_4_] + [NaPO_4_^2−^] + [NaHPO_4_^−^] + [KPO_4_^2−^] + [KHPO_4_]) (10)

Thus, [H_2_PO_4_^−^]/Σ[Pi] is the calculated fraction of Pi that is in the second (of three) protonation states of Pi (Figure 2D–F and Equation (9)). In Equation (9), pK_a_ is the negative log of the acid dissociation constant at 20 °C, *d*pK_a_/*d*T is the change in pK_a_ per °C, and *A* is a scaling factor. All calculated values of [H_2_PO_4_^−^]/Σ[Pi] at pMg 2 (points in Figure 2D) were simultaneously fitted to Equation (9); the regression predictions are shown as lines in Figure 2D and the fit parameter estimates are provided in Table 1. This was repeated twice more for pMg 3 (Figure 2E and Table 1) and pMg 4 (Figure 2F and Table 1). As is evident in Figure 2, the data (left panels) and calculations (right panels) follow similar trends, although the fit parameters (Table 1) indicate a slightly greater variation of pK_a_ with [Mg^2+^] and temperature than predicted.

### 2.2. Determination of the Equilibrium Constant for the Creatine Kinase Reaction from HPLC Analyses of Solutions

#### 2.2.1. Apparent Equilibrium Constant K_eq_″

**Figure 3:**
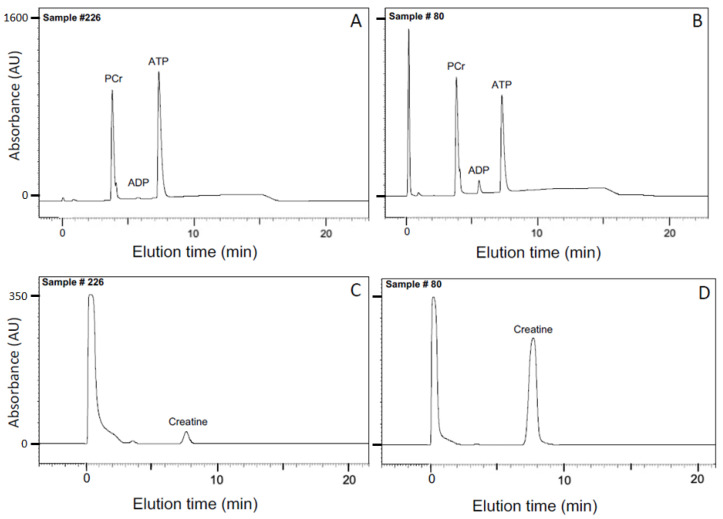
HPLC quantitation of (**A**,**B**) PCr, ADP, and ATP by anion exchange chromatography and (**C**,**D**) Cr by cation exchange chromatography (Section 4.3) in solutions designed to mimic the cytosol under different metabolic conditions (Section 4.1). Representative sample #226 (panels (**A**,**C**); low ADP; no added Cr) was held at 10 °C, pMg 4 and pH 8. Representative sample #80 (panels (**B**,**D**); high ADP; 50 mM added Cr) was held at 40 °C, pMg 2 and pH 8. Both samples initially contained 1 mg/mL rabbit CK. At equilibrium as determined by monitoring the reactions using ^31^P-NMR spectroscopy (Figure 1), each reaction was stopped with addition of SDS at 2% per mg CK (Section 4.2). For both HPLC methods, detection was by optical absorbance at 210 nm and peak areas were quantified against calibration curves determined using known standards. Numerical scales on vertical (optical absorbance) axes correspond to detector output in millivolts. Note that scales for the vertical (optical absorbance) axes are the same for panels (**A**,**B**) and for panels (**C**,**D**).

After equilibrium was demonstrated by ^31^P-NMR (Figure 1), the CK reaction was terminated by denaturing the enzyme, followed by HPLC analysis of metabolites (Section 4.3); SDS was chosen as the denaturant (Section 4.2) to minimize spontaneous hydrolysis of analytes that occurs with some other methods of stopping enzyme-catalyzed reactions. Representative chromatograms for two conditions in the large matrix of solutions (Section 4.1) are shown in Figure 3: pMg 4.0, pH 8, and 10 °C (Figure 3A,C) and pMg 2.0, pH 8, and 40 °C (Figure 3B,D). These two conditions represent low or high concentrations, respectively, for both ADP (anion exchange chromatography in Figure 3A,B) and Cr (cation exchange chromatography in Figure 3C,D).

To optimize estimation of apparent equilibrium constants using regression analysis on HPLC data, we reformulated Equation (6) (K_eq_′) to incorporate pH, i.e., to bring it closer to K_eq_ (Equation (5)). While K_eq_″ (Equation (11)) includes pH, it retains compatibility with the analytical measurements of metabolites where the individual species are not distinguished experimentally:

(11) $$K_{eq}^{\prime \prime }\left[pMg,T\right]=\frac{\sum \left[Cr\right]\sum \left[ATP\right]}{{\left(10^{-pH}\right)}^{\beta }\sum \left[PCr\right]\sum \left[ADP\right]}$$

Rearrangement of Equation (11) yields a form that is suitable for nonlinear regression analysis on the aggregate of the HPLC data obtained for the family of solutions (all pH values) at a single pMg and temperature:

(12) $$\sum \left[ADP\right]=\frac{\sum \left[Cr\right]\sum \left[ATP\right]}{K_{eq}^{\prime \prime }\left[pMg,T\right]{\left(10^{-pH}\right)}^{\beta }\sum \left(PCr\right)}$$

**Figure 4:**
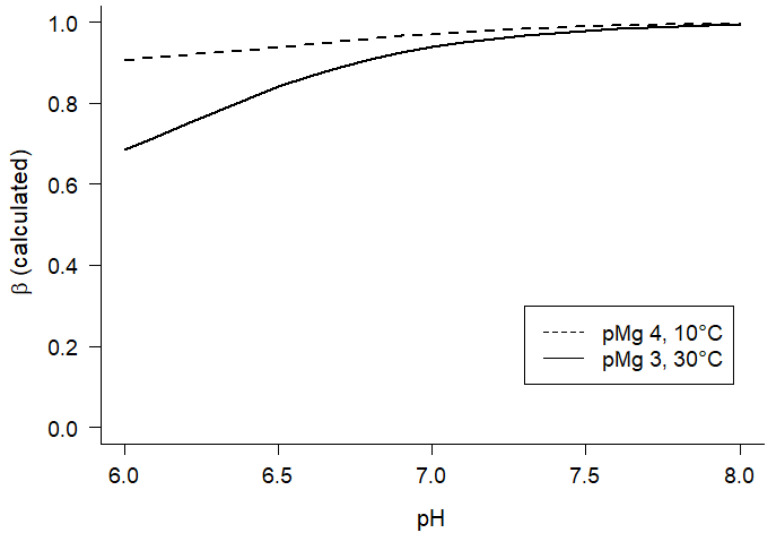
Calculated pH dependence and range of the stoichiometric coefficient of proton consumption (β) by ADP rephosphorylation via transfer of Pi from PCr (Equation (4)). pH dependence of β was predicted according to the ion binding equilibria (Section 4.1) for all conditions of this study. The smallest range of predicted values as a function of pH was obtained at pMg 4.0 and 10 °C (dashed line), while the largest range of predicted values as a function of pH was obtained at pMg 3.0 and 30 °C (solid line); all other predicted values of β fell within the range between the two lines shown.

However, in order to obtain the desired estimates of K_eq_″ using Equation (12) it was necessary to evaluate β, which is the net proton stoichiometric coefficient for hydrolysis of PCr to Cr (Equations (5), (11), and (12)). Therefore, we estimated β by calculating values for each of the experimental conditions according to the approach employed for constructing solutions (Section 4.1). For each combination of temperature and pMg, the calculated β was <1.0 at pH 6 and β → 1 as pH increased from 6 to 8 (Figure 4), which is in general agreement with previous estimates [56,57]. Of all of the conditions examined in this study (Section 4.1), the two conditions included in Figure 4 illustrate the smallest (10 °C, pMg 4, dashed line) and largest (30 °C, pMg 3, solid line) variations in β calculated over the pH range of 6–8.

**Figure 5:**
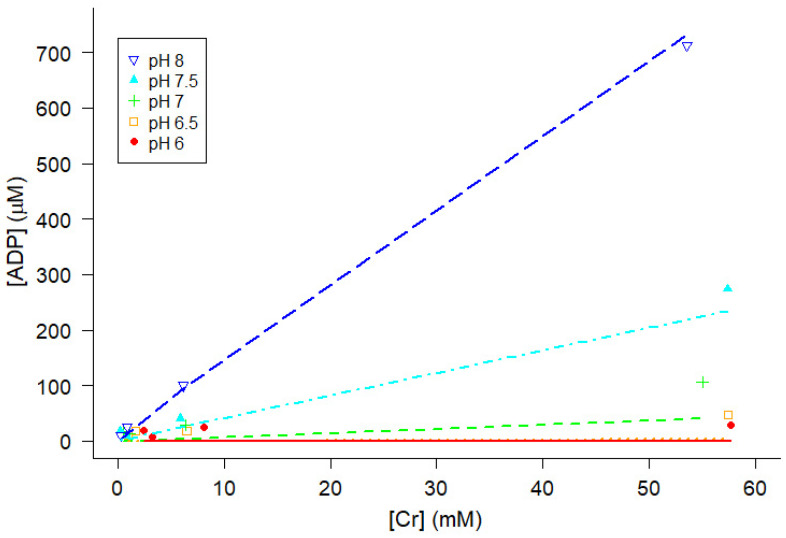
Nonlinear least squares regression estimation of K_eq_″ for model solutions at pMg 3.0 and 30 °C. Total concentrations of ADP, ATP, Cr, and PCr were measured by HPLC (Figure 3), although only ADP and Cr are plotted here because they vary most widely by the amount of Cr added. All data (all Cr and pH values) were used to simultaneously obtain a single estimate of K_eq_″ [pMg 3.0, 30 °C] by nonlinear least squares regression fitting of the data to Equation (12) using values of β, as illustrated in Figure 4. Colors correspond to pH (blue, pH 8; cyan, pH 7.5; green, pH 7; orange, pH 6.5; red, pH 6). Comparable analyses were performed for each combination of temperature and pMg, resulting in a total of 12 plots (Figure A1) and regression estimates of K_eq_″ [pMg, T] (Figure 6).

At a given temperature, chromatographic analyses of pMg and pH showed that ADP increased as the amount of Cr added increased and that ADP increased as pH increased at a given temperature, pMg, and Cr (Figure 5). Simultaneously fitting all of the data at 30 °C and pMg 3 for all pH values to Equation (12), the regression estimate of K_eq_″ was 1.075 × 10^9^ M^−1^ ± 0.036 × 10^9^ (Figure 5). For the purposes of this study, it was sufficient to obtain a regression estimate of K_eq_″ at each of the combinations of pMg and temperature (twelve total combinations yielding twelve estimates of K_eq_″ [pMg, T]), as the major goal of these measurements was to estimate [ADP] in experiments where it is difficult to measure [ADP] directly, thereby enabling calculation of ΔG_ATP_). HPLC data for all twelve combinations of temperature and pMg were independently fitted to Equation (12) (Appendix A) to obtain a matrix of estimates of K_eq_″ [pMg, T].

#### 2.2.2. Dependence of K_eq_″ for the Creatine Kinase Reaction on Mg^2+^ and Temperature

**Figure 6:**
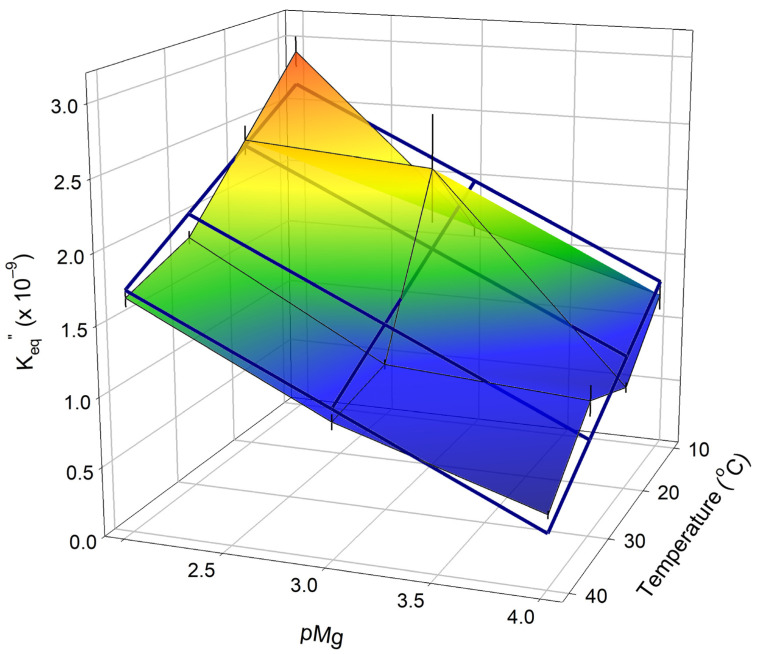
Creatine kinase K_eq_″ as a function of free Mg^2+^ (pMg) and temperature at Γ/2 = 0.25 M. Points are regression estimates of K_eq_″ (Equations (10) and (11)) obtained as illustrated (Figure 5 and Figure A1). Error bars are the SE regression for each estimate of K_eq_″. The results of a multiple linear least squares regression on all of the K_eq_″ data (Equation (13)) are shown by thick blue lines. This simple relationship allows for prediction of [ADP] (which is not always directly measurable, i.e., as illustrated in Figure 1 and Figure 3) over a wide range of conditions, ranging from those relevant to intact tissue in vivo to experiments with permeabilized muscle fibers.

**Figure A1:**
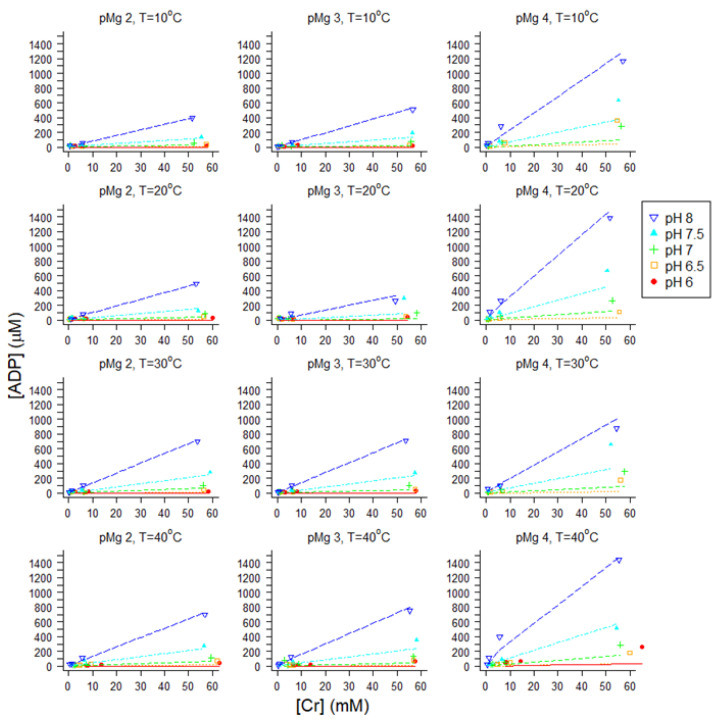
Nonlinear least squares regression estimation of K_eq_″ [pMg, T] for all twelve sets of conditions employed in this study. Total concentrations of ADP, ATP, Cr, and PCr were measured by HPLC (as illustrated in Figure 3), although only ADP and Cr are plotted here because they vary most widely due to the amount of Cr added. K_eq_″ [pMg, T] was obtained by fitting each dataset to Equation (12) using values of β, as illustrated in Figure 4 (data for the pMg 3.0 and 30 °C condition are plotted in Figure 5). Within each panel, the colors correspond to pH (blue, pH 8; cyan, pH 7.5; green, pH 7; orange, pH 6.5; red, pH 6). The panel columns correspond to free Mg^2+^ (left-to-right: pMg 2.0, pMg 3.0, pMg 4.0) and the panel rows correspond to the temperature (top-to-bottom: 10 °C, 20 °C, 30 °C, 40 °C). Axis scales were kept the same in all panels for ease of comparison among conditions. All data (all Cr and pH values) at each combination of constant pMg and temperature were used to simultaneously obtain one estimate of K_eq_″ [pMg, T]. A summary of the results of these analyses (K_eq_″ [pMg, T]) is shown in Figure 6.

Nonlinear regression parameter estimates of CK K_eq_″ (Equation (12)), obtained as shown in Figure 5 and Figure A1, are shown in Figure 6 as a 3D surface plot. Multiple linear regression was performed to obtain a simple predictive equation for K_eq_″ [pMg, T] over the entire matrix of conditions employed:

(13) $$K_{eq}^{\prime \prime }\left[pMg,T\right]=\left(\left(-0.67\pm 0.11\right)pMg+\left(-0.031\pm 0.008\right)T+\left(4.3\pm 0.4\right)\right)\times 10^{9}$$

where T is the temperature in °C and the three regression parameter estimates (in parentheses) are provided ± SE regression (multiple R^2^ = 0.855). Predictions from the multiple regression (Equation (13)) are shown in Figure 6 connected by thick blue lines. The empirical relationship in Equation (13) can be used in combination with Equation (12) to obtain estimates of cytoplasmic ADP levels over the broad physiologically and experimentally relevant range of pH 6–8, pMg 2–4, and T = 10–40 °C. This result is useful on its own for experiments on living cells using results that are typically measured in bioenergetic experiments, such as ^31^P-NMR spectroscopy in combination with chemical analysis.

### 2.3. Estimation of *Δ*G_ATP_

To determine ΔG_ATP_ for evaluation of mechanical measurements under various biochemical conditions, we evaluated the following relationship, which is a more complete description of Equation (3) that takes into account pH and ion binding equilibria (Section 2.1):

(14) $${∆G}_{ATP}=-RT\ln\left(\left(\frac{f_{ATP}}{f_{ADP}\;f_{Pi}}\right)\frac{K_{ATP}\left[ATP\right]}{\left[ADP\right]\left[Pi\right]\left[H^{+}\right]}\right)$$

where *f*_ATP_, *f*_ADP_, and *f*_Pi_ are:

(15) $$\begin{array}{c} f_{ATP}=\frac{\left[{ATP}^{4-}\right]}{\sum \left[ATP\right]}\\ f_{ADP}=\frac{\left[{ADP}^{3-}\right]}{\sum \left[ADP\right]}\\ f_{Pi}=\frac{\left[{HPO}_{4}^{2-}\right]}{\sum \left[Pi\right]} \end{array}$$

All of the ratios in Equation (15) vary with [H^+^], [Mg^2+^], and temperature, and can be calculated from the equilibrium binding constants as described for the solution calculations (Section 2.1).

Equation (14) can be expanded to a form that is more useful for calculating the individual contributions of each component:

(16) $${∆G}_{ATP}=-RT\ln K_{ATP}-RT\ln\left(\frac{f_{ATP}}{f_{ADP}\;f_{Pi}}\right)+RT\ln\left[H^{+}\right]+RT\ln\left[ADP\right]+RT\ln\left[Pi\right]\;-RT\ln\left[ATP\right]$$

The first three terms in Equation (16) comprise ΔG^0^_ATP_ (Equation (3)), which together account for the pH and Γ/2 dependencies of ΔG_ATP_ as well as part of the temperature dependence. K_ATP_ was set to 9.91 × 10^−7^ M^2^, meaning that ΔG^0^_ATP_ was −32 kJ mol^−1^ at pH 7, pMg 3, and 37 °C [29]. Values for [ADP] were calculated from Equation (12) using K_eq_″ from Equation (13) with the values of [ATP], [PCr], [Cr], and [H^+^] measured in each solution. Note that pH, [Mg^2+^], and temperature each affect ΔG_ATP_ nonlinearly in Equation (16). For example, ΔG_ATP_ varies with pH because of the proton concentration term (RT ln[H^+^]) and because pH affects the ratios *f*_ATP_, *f*_ADP_, and *f*_Pi_ (Equation (15)). In addition, there is an influence of pH on K_eq_″ that markedly alters [ADP] at given levels of [ATP], [PCr], and [Cr] (Figure 5 and Figure A1).

### 2.4. Influence of [Pi] on *Δ*G_ATP_ and Muscle Force

**Figure 7:**
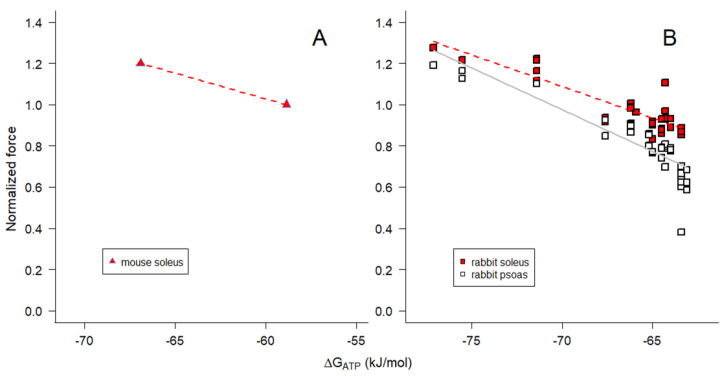
Variation of maximum Ca^2+^-activated force with ΔG_ATP_ when ΔG_ATP_ was altered by varying [Pi] in (**A**) intact mouse soleus muscles (red filled triangles with blue borders and with dashed red line) or (**B**) single skinned fibers from rabbit psoas (open squares with solid gray line) and soleus (red filled squares with dashed red line) muscles. [Pi] was varied in intact muscles (panel (**A**)) by altering the substrate supplied extracellularly [49], and the force and biochemical data are from that study; [Pi] in the presence of pyruvate was set to 1 mM in the plot, with the upper limit determined [49]. [Pi] for skinned fiber experiments (panel (**B**)) was 0.1–36 mM. The psoas force at the first Pi concentration in panel (**B**) was previously published in Chase and Kushmerick [35], and the psoas force at the second Pi concentration in panel (**B**) was previously published in Chase and Kushmerick [36]; all other data in panel (**B**) are previously unpublished. Measurements on skinned fibers in panel (**B**) were from N = 28 psoas fibers from ten rabbits and N = 19 soleus fibers from six rabbits. As described in the text, the force for the psoas fibers in panel (**B**) was normalized to 1 mM Pi, while the soleus force was normalized to 6 mM to reflect the higher basal levels of Pi in slow muscle [58]. The points in panel (**A**) are mean values, while the points in panel (**B**) are individual measurements. The regression slopes are −0.0248 in panel (**A**), −0.0306 (regression SE 0.0029) for soleus data in panel (**B**), and −0.0406 (regression SE 0.0038) for psoas data in panel (**B**); note that the slopes are not significantly different for soleus data in panels (**A**,**B**), while the slope for skinned psoas fibers is significantly steeper than that for skinned soleus fibers.

Within living skeletal muscle, the cytoplasmic Pi concentration can vary over a wide range [37,58]. In permeabilized muscle, logarithmic increases in [Pi] depress the maximum Ca^2+^-activated isometric force [45,59,60]. Taken together, these observations suggest that within rather wide and physiologically relevant limits the force likely varies linearly with ΔG_ATP_ (Equation (3)), at least with respect to variations in Pi concentration. To quantitatively examine this possibility, we first examined tetanic force of isolated soleus muscle from mice in the presence and absence of pyruvate in the bathing medium (Figure 7A). In the presence of pyruvate, the intracellular Pi of slow muscle is reduced from approximately 6 mM (control) to 1 mM or less, as determined by ^31^P-NMR spectroscopy [49]. Force for isolated soleus muscle was normalized to the control condition. Fast muscle results are not included because the resting Pi is much lower (~1 mM or less) compared with slow muscle [49,58], and we could not discern through our methods whether addition of pyruvate reduced intracellular Pi substantially enough to influence isometric force.

**Figure A2:**
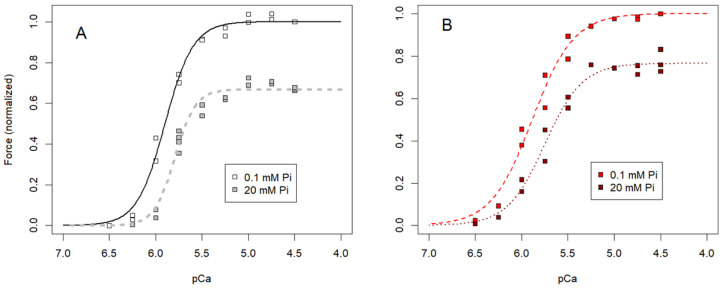
Isometric force–pCa relationships for single permeabilized fibers from (**A**) rabbit psoas and (**B**) rabbit soleus muscles (Section 4.4.1) at 0.1 mM Pi (open squares and solid line in panel (**A**); red squares and dashed line in panel (**B**)) or 20 mM Pi (gray squares and dotted line in panel (**A**); dark red squares and dotted line in panel (**B**)). Measurements are from N = 4 psoas fibers from two rabbits in panel (**A**) and N = 3 soleus fibers from two rabbits in panel (**A**). Not shown are data points at pCa ≥ 8 that corresponded to normalized force = 0, as the absolute value (passive force) for each fiber was subtracted from all force values before plotting. Parameter estimates for nonlinear regressions on the Hill Equation (Equation (A2)) are provided in Table A1.

In order to allow variation of [Pi] beyond what is possible in vivo, we measured the maximum steady-state isometric force of single skinned fibers from rabbit soleus (Figure 7B, red squares) and psoas (Figure 7B, open squares) muscle when [Pi] in the bathing solution was varied between 0.1 and 36 mM. This range was the maximum extent of variation that could be achieved without exceeding the ionic strength constraint (Section 4.4.1) on the upper end of the Pi concentration range or adding a Pi “mop” [45,59,61] to extend the lower end. We verified that sufficient activating Ca^2+^ was present to achieve maximum force despite the decrease in Ca^2+^-sensitivity (rightward shift of the force–pCa relation) observed at elevated Pi levels in both psoas (Figure A2A and Table A1) and soleus (Figure A2B and Table A1) muscle fibers. Our observation of decreased Ca^2+^-sensitivity with elevated Pi is consistent with previous observations by others [62,63,64,65,66]. Each data point in Appendix C was normalized to bracketing control measurements at 0.1 mM Pi. The force for each skinned psoas fiber (Figure 7B) was renormalized to the regression estimate of force at 1 mM Pi, a concentration that is comparable to what is found in living fast muscle fibers [58]. Force for skinned soleus fibers (Figure 7B) was renormalized to the regression estimate of force at 6 mM Pi for consistency with the isolated soleus muscle data (Figure 7A).

**Figure A3:**
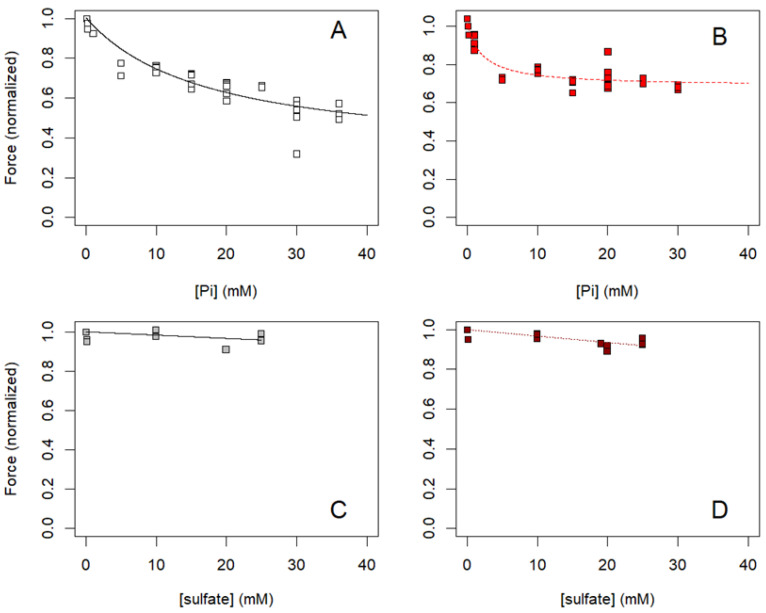
Dependence of isometric force on (**A**,**B**) [Pi] and (**C**,**D**) [sulfate] for single permeabilized fibers from (**A**,**C**) rabbit psoas and (**B**,**D**) rabbit soleus muscles (Section 4.4.1). Each point represents the normalized maximum force (normalized as described for the data in Figure A2) for one muscle fiber at one concentration of Pi (**A**,**B**) or sulfate (**C**,**D**). Measurements are from N = 28 psoas fibers from ten rabbits in panel (**A**), N = 19 soleus fibers from six rabbits in panel (**B**), N = 7 psoas fibers from one rabbit in panel (**C**), and N = 7 soleus fibers from one rabbit in panel (**D**). The lines in panels (**A**,**B**) were fitted to Equation (A3) using nonlinear least squares regression, while the lines in panels (**C**,**D**) were fitted using linear least squares regression constrained to pass through normalized force = 1.0 at [sulfate] = 0.

For all three muscle preparations, the maximum isometric force decreased with increasing Pi (Figure 7, Figure A2 and Figure A3A,B), which corresponds to the force decreasing as ΔG_ATP_ became less negative (Figure 7). ΔG_ATP_ was calculated for each experimental condition according to Equation (16), assuming that the CK reaction (Equation (4)) was at equilibrium in the muscle preparations. The slopes for the three relationships between force and ΔG_ATP_ were linear, and were similar for intact and skinned soleus preparations over the experimental ranges examined. Note that the skinned fiber and isolated muscle datasets from soleus muscles are offset on the horizontal axes in Figure 7 because the skinned fiber conditions (primarily the levels of Cr and ADP and the temperature) were not designed to exactly match the conditions in living muscle cytoplasm.

The [Pi]-dependence of isometric force for permeabilized fibers from rabbit psoas (Figure A3A) and soleus (Figure A3B) muscles illustrates that slow fibers are more sensitive to Pi in the sense that the force declines to a greater extent at lower concentrations of Pi. Considering this observation in the context of physiological levels of intracellular Pi, where fast muscle has much lower levels of cytoplasmic Pi at rest [58], the data in Figure 7 and Figure A3A,B indicate that the maximum isometric force of fast muscle should be higher than that of slow muscle in healthy living muscles. Control experiments where sulfate concentration was varied at a constant baseline of 0.1 mM Pi showed that [SO_4_^=^] caused only a small decline in isometric force in permeabilized fibers from both fast (Figure A3C) and slow (Figure A3D) muscles relative to that observed over the same concentration range of Pi (Figure A3A,B). Thus, the inhibitory effects of Pi on isometric force are not due to nonspecific effects of multivalent anions, and the variation of force with ΔG_ATP_ when [Pi] is varied (Figure 7) can be directly attributed to the contribution of [Pi] to ΔG_ATP_.

**Figure A4:**
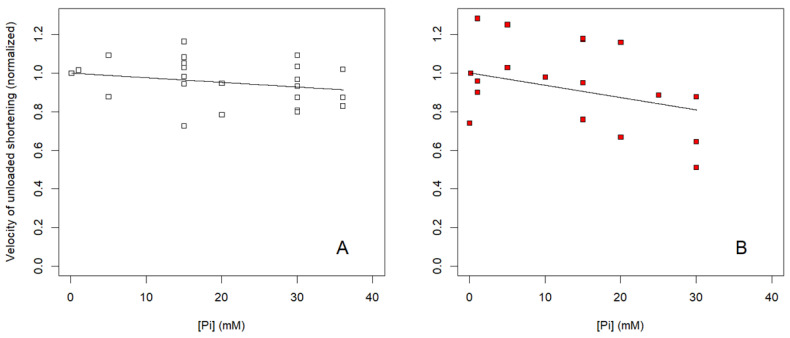
Dependence of the velocity of unloaded shortening (V_US_) on [Pi] for single permeabilized fibers from (**A**) rabbit psoas (open squares) and (**B**) rabbit soleus muscles (red squares) (Section 4.4.1). Each point represents the normalized value of V_US_ (normalized to V_US_ obtained at 0.1 mM Pi in the same fiber, comparable to normalization of force data as described in Figure A2 and Figure A3) at maximum Ca^2+^-activation. Measurements are from N = 18 psoas fibers from eight rabbits in panel (**A**) and N = 9 soleus fibers from six rabbits in panel (**B**). The control values of V_US_ were 3.3 ± 0.8 FL s^−1^ (mean ± SD) for psoas fibers and 0.7 ± 0.2 FL s^−1^ (mean ± SD) for soleus fibers. The lines are linear least squares regression fits constrained to pass through V_US_ = 1 at 0.1 mM Pi.

Our examination of the relationship between unloaded shortening velocity (V_US_) and Pi (Figure A4) confirmed prior studies that showed Pi to have little or no effect on the rate limiting step for unloaded shortening [47,48,52]. This means that, in contrast to isometric force (Figure 7 and Figure A3A,B), ΔG_ATP_ does not directly influence V_US_ in skeletal muscle.

### 2.5. Influence of pH on *Δ*G_ATP_, [ADP], and Muscle Force

In view of the strong relationship between the maximum Ca^2+^-activated isometric force and ΔG_ATP_ when [Pi] is varied (Figure 7), we extended the investigation by re-examining previously published measurements made with isolated perfused cat muscles [67] and skinned fibers from rabbit psoas and soleus muscles [35] when the pH surrounding the myofilaments was varied. In the study by Harkema et al. [67], intracellular acidification of biceps and soleus muscles was achieved by perfusion with a hypercapnic perfusate, and pH was determined by ^31^P-NMR in a manner comparable to that shown in Figure 1; force was then normalized to the normocapnic condition. In the study on single skinned fibers from rabbit muscles, the maximum steady-state isometric force was measured using psoas and soleus fibers when the pH of the bathing solution was varied between pH 6 and 8 [35]; the force for each skinned fiber was normalized to the pH 7.1 condition, which is comparable to that in living fast and slow muscles.

**Figure 8:**
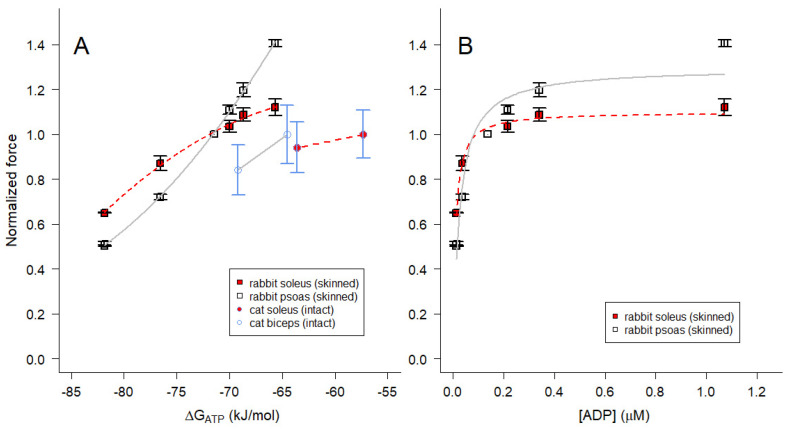
Maximum Ca^2+^-activated isometric force variation with (**A**) ΔG_ATP_ and (**B**) [ADP] when pH was altered. We reanalyzed previously published data from (panel (**A**)) isolated perfused biceps (open circles with blue error bars connected by solid gray line) and soleus (red filled circles with blue error bars connected by red dashed line) muscles from cat [67] and (panels (**A**,**B**)) single skinned fibers from rabbit psoas (open squares fit with solid gray lines) and soleus (red filled squares fit with dashed red lines) muscles [35]. Hypercapnic (acidic cytoplasmic pH 6.48–6.6) force data from cat muscles were normalized to the normocapnic condition (higher force; cytoplasmic pH 7.09–7.11) for each muscle type. Skinned fiber force data were normalized to that at pH 7.1 for the same fiber. Lower forces were associated with acidic (lower) pH and higher forces were associated with basic (higher) pH [35,67]. The points represent the average ± SD. ΔG_ATP_ was estimated according to Equation (16) and [ADP] was estimated according to Equation (12) using values of K_eq_″ from Equation (13). In panel (**A**) smooth curves were drawn through the points, while in panel (**B**) the lines represent nonlinear regression fits to Equation (17) (regression parameter estimates for *K_m_* are provided in the text). Note that in panel (**A**) the slope is steeper for fast muscles than for slow muscles when comparing within a species.

To examine the dependence of the isometric force on free energy when pH was varied, ΔG_ATP_ was calculated for each experimental condition (Equation (16)), assuming that the CK reaction was at equilibrium. Force declined and ΔG_ATP_ became more negative with decreased pH in both living and permeabilized muscles (Figure 8A). The slopes for the skinned fiber relationships between force and ΔG_ATP_ were nonlinear and the slopes were positive for all preparations, opposite to what was observed when Pi was varied (Figure 7). In both intact and skinned muscles, the slope for fast fiber types was steeper than that for slow fiber types (Figure 8A). We conclude that the variation in ΔG_ATP_ with pH (Figure 8A), in contrast to Pi (Figure 7), was not due to any direct influence of pH on ΔG_ATP_.

ΔG_ATP_ varies with pH in part because of a direct contribution of [H^+^] in Equation (16) and in part because it influences the terms in Equation (16) containing *f_ATP_*, *f_ADP_*, and *f_Pi_* (Equation (15)) as well as [ADP] (Equation (12) and Figure 5). In particular, [ADP] at acidic pH is reduced to very low levels, on the order of 10 nM at pH 6.0 in the experiments described here (Equation (12) and Figure 5) and lower than what was attained in our prior experiments examining the effects of ADP on skeletal muscle contractility [36]. Therefore, we examined the relationship between the steady-state isometric force and [ADP] (Figure 8B). The isometric force data for both fast and slow fiber types were described by a saturable binding relation (Equation (17)), with the affinity constants (K_m_) estimated by nonlinear least squares regression (±SE) of 24.0 ± 5.3 nM for psoas fibers and 9.0 ± 0.9 nM for soleus fibers (Figure 8B). These values are consistent with the lack of effect of higher concentrations of ADP on isometric force at pH 7.1 reported in Chase and Kushmerick [36], and would be slightly lower if we considered only the proportion of ADP in the Mg^2+^-bound form (MgADP). However, it seems likely that protons modulate force by additional mechanisms beyond altering [ADP].

(17) $$\frac{F\left(pH\right)}{F\left(pH\;7.1\right)}=\frac{A\left[ADP\right]}{K_{m}+\left[ADP\right]}$$

## 3. Discussion

The main results of this study are three-fold. First, we established a comprehensive formalism relating the apparent equilibrium constant (K_eq_″) for the creatine kinase reaction to broad changes in critical components, specifically Mg^2+^ and temperature, that differ within and between experimental preparations and protocols. In circumstances where the CK reaction is at equilibrium, these factors, along with pH, influence two parameters, namely, cytosolic ADP and ΔG_ATP_, in a predictable manner—even though they typically cannot be measured directly—when [ATP], [Pi], [PCr], and [Cr] have been determined. Second, these results were applied to calculate ADP and ΔG_ATP_ for experimental conditions in which the biochemical conditions were known and mechanical measurements could be made in skeletal muscle preparations. Third, we found that there appears to be marked variability in the contractile efficiency of force generation by skeletal muscle with changes in energetic conditions due to altered [Pi] or pH.

### 3.1. Estimation of K_eq_″ for the Creatine Kinase Reaction and Cytoplasmic Free ADP

The results of the first portions of our analysis allow quantitative estimation of intracellular pH from ^31^P-NMR spectra (Figure 2 and Table 1) and K_eq_″ for the CK reaction (Figure 6 and Equation (13)) and ADP (Figure 5 and Equation (12)) from a combination of chemical and ^31^P-NMR assays over a considerably wider range of physiological and biochemically relevant conditions than previously examined experimentally.

Our results on the use of δ_Pi_ from ^31^P-NMR spectra to estimate pH_i_ (Figure 2, Equation (8), and Table 1) are in good agreement with the approach of Kost [55] over a similar range of temperatures, and extend the analysis over a wider range of Mg^2+^ concentrations, a value that can be determined experimentally [30,68,69,70]. Data from these titration curves (Figure 2) are quite useful for in vivo ^31^P-NMR studies at and beyond 37 °C. These curves were produced without incidental modulation of the solution, as occurs, e.g., during traditional titrations that incrementally add acid or base, thereby changing Γ/2. Thus, we avoided any influence on the chemical shift endpoints for Pi (the extreme acid δ_A_(T) and basic δ_B_(T) chemical shifts) or the pK_a_ of Equation (8) [55].

The concept of determining K_eq_′ for the CK reaction en route to estimation of cytoplasmic [ADP] is well established [29], although its applicability has previously been limited to narrow ranges of conditions (note that K_eq_′ defined in Equation (6) applies to a specific pH, in contrast to K_eq_″ defined in Equation (11)). K_eq_′ for the CK reaction reported by Lawson and Veech [29] for physiologic conditions of 37 °C and pH 7.0 has been widely used, often with adjustments necessary for experimental temperature and/or pH. Lawson and Veech [29] evaluated the dependence of K_eq_′ on [Mg^2+^] over a wider range of [Mg^2+^] concentrations than reported here, though at a constant pH 7. They varied pH (pH ~ 7–8) over a limited range of [Mg^2+^]; however, utilizing these broader ranges of conditions to calculate ADP typically requires estimation, interpolation, and in many instances, extrapolation.

The effect of temperature (5–38 °C) on the observed K_eq_′ for the creatine kinase reaction at pH ~7 has been reported from empirical studies [71], showing that CK K_eq_′ increases as temperatures decreases. This is in agreement with the data in Figure 6 and the corresponding negative regression coefficient for the temperature term in Equation (13). Further theoretical work extrapolated values for CK K_eq_′ as a function of both temperature and ionic strength [72]. Golding et al. [73] calculated that at 38 °C, K_eq_′ increases when pH or pMg decrease. The former agrees with the expectations from Equations (6) and (10), and the latter is consistent with the data in Figure 6 and the corresponding, negative regression coefficient for the pMg term in Equation (13). However, Golding et al. [73] did not include binding constants for important cations known to be present in the cytosol, including K^+^ and Ca^2+^, presumably because of their impact on proton binding coefficients, making extrapolation to these extremes difficult to interpret [74].

Considering the dependence on interpolation and extrapolation from experimental measurements to obtain an estimate of CK K_eq_′ along with the potential for wide-ranging estimates of [ADP], we decided that a comprehensive strategy was necessary to generate a comprehensive set of empirically derived values for CK K_eq_′ (ultimately K_eq_″). This strategy involved the construction of a matrix of model solutions utilizing binding constants and enthalpic terms for metabolite binding of all important ions present within the cytosol, sensitive analytic methods to determine metabolite contents for calculation of the equilibrium values for each condition, and a statistical approach to derive coefficients for proton stoichiometry over the entire data matrix. The calculations that went into constructing solutions such as those used in our experiments are well-established and have been received considerable attention and research effort [35,44,64,74,75,76,77]. As is evident in these references, a primary focus is to use these solutions to mimic major (though not all) specific aspects of the intracellular milieu in experiments on permeabilized muscle.

The results in Figure 6, along with the regression results in Equation (13), allow for reliable estimation of K_eq_″ across the pH range of 6.0–8.0, pMg range of 2.0–4.0, and temperature range of 10–40 °C. From this, [ADP] can be estimated using Equation (12) for given conditions of [ATP], [PCr], [Cr], [Mg^2+^], pH, and temperature when CK is present with sufficient activity to achieve equilibrium. This appears to be the best approach for obtaining estimates of [ADP] under physiological conditions, and would be useful for studies on striated [37,57,58] and smooth [78] muscles. A FRET biosensor for ADP has been developed [79]; however, it cannot be expressed in vivo because its synthesis includes covalent modification of the protein component with rhodoamine fluorescent labels. Perhaps a FRET biosensor for ATP that can be expressed in cells [80] could be altered to discriminate physiologically relevant levels of ADP in the presence of the much higher levels of ATP found in healthy cells.

### 3.2. Estimation of *Δ*G_ATP_

The results described in the previous section greatly expand the range of physiological and experimental conditions for which ΔG_ATP_ can be more easily and reliably estimated based on direct measurements of parameters that are part of many experimental routines. Longstanding studies of Alberty and co-workers, as well as others, have provided calculations for estimating ΔG_ATP_ under a wide variety of conditions [23,25,81,82,83,84,85,86]. To apply the results of these studies to living tissues, however, requires knowledge of cytoplasmic [ADP] in addition to [ATP], [Pi], pH, [Mg^2+^], etc. Thus, this extensive body of valuable work on its own is not sufficient to estimate cellular ΔG_ATP_.

We estimated ΔG_ATP_ to be −58.9 kJ mol^−1^ for mouse soleus (slow) muscle, with glucose as substrate at 25 °C (Figure 7A), and to be −57.3 kJ mol^−1^ for cat soleus (slow) muscle and −64.6 kJ mol^−1^ for cat biceps (fast) muscle with normocapnic perfusate at 37 °C (Figure 8A). The difference between the slow and fast muscle types stems largely from the higher levels of Pi in slow muscles at rest, though there is a contribution from slightly lower ATP levels in slow muscles as well [49,58,67]. Perhaps surprisingly, differences in PCR and Cr contribute little to the fiber type difference in ΔG_ATP_; the resulting ADP levels due to the CK reaction are not very different (15.8 μM for mouse soleus, 27.6 μM for cat soleus, and 16.3 μM for cat biceps). The implications of these results impact the precise calculation of free [ADP] to values that in certain circumstances may be lower than previously calculated, necessitating the reinvestigation of previously reported ADP-dependent processes.

ΔG_ATP_ values from our skinned fiber experiments were substantially more negative than those from intact muscles of the same fiber type (Figure 7 and Figure 8A). We estimated ΔG_ATP_ to be −71.4 to −71.5 kJ mol^−1^ for permeabilized fibers from rabbit psoas (fast) muscle at 1 mM Pi and 12 °C (psoas controls in Figure 7B and Figure 8A, respectively). ΔG_ATP_ for permeabilized soleus fibers would be exactly the same for the same solution conditions (e.g., −71.4 kJ mol^−1^ for soleus control in Figure 8A), although ΔG_ATP_ was less negative for the soleus control in Figure 7B because force normalization in the [Pi] experiments accounted for the higher basal [Pi] in that fiber type [49,58,67]. A substantial contributor to the more negative values of ΔG_ATP_ for permeabilized muscles is the much lower [ADP] (~2 orders of magnitude) due to lesser amounts of Cr (also ~2 orders of magnitude, per Equation (12)) present in the control conditions for skinned fibers (Figure 8B) [36].

### 3.3. Implications for Actomyosin Interactions and the Physiology of Skeletal Muscle

The results of this study allowed us to make initial steps toward quantifying the relationship between the maximum Ca^2+^-activated isometric force and available energy over a wide range of conditions in situations where all of the relevant parameters can be controlled and/or measured. ATP plays two roles in the actomyosin crossbridge cycle: binding of MgATP to a nucleotide-free (rigor) crossbridge results in rapid dissociation of the myosin head from the thin filament; then, ATP hydrolysis by the myosin head (Equation (2)) provides the energy for the mechanical power stroke in the next crossbridge cycle. ΔG_ATP_ provides the ultimate limit for work performed by actomyosin [38,40,45,87]. The dependence of actomyosin function on ΔG_ATP_ is mechanistically important for understanding the energetics of actomyosin’s ATPase cycle and for assessing physiological changes during hypoxia and muscle fatigue, where there are substantial alterations in cytosolic metabolite concentrations and cellular energy status.

Our data in Figure 7 and Figure A3A,B are consistent with other studies on skinned muscle fibers in which the maximal steady-state isometric force varies logarithmically with [Pi] over a wide concentration range, including the physiological range of [Pi] [44,46,47,48,52,60,64,65,88,89,90]. However, force for fast skeletal muscle fibers plateaus below ~100 μM [45,59,91], a concentration range that we did not explore (Figure 7 and Figure A3A,B). Thus, within the limits of our experimental measurements, the data in Figure 7 are consistent with the ΔG_ATP_ limiting force when [Pi] is varied (the second to last term on the right side of Equation (16)) according to the description of Pate et al. [92].

Our [Pi] data, when plotted on a linear scale (Figure A3A,B), are in apparent agreement with others suggesting that slow fibers are more sensitive to Pi compared with fast fibers at low Pi concentrations, but are less sensitive to Pi at high concentrations [48,88,93]. The latter is consistent with the difference in slopes when force is plotted against ΔG_ATP_ when [Pi] is varied (Figure 7B), which effectively corresponds to plotting on a logarithmic axis for [Pi]. The slopes in Figure 7B are significant because of their relation to the energetics of actomyosin interactions [45,59,92]. Interestingly, the slope obtained with intact soleus from mouse appears to be similar to that obtained with skinned fibers from rabbit soleus (Figure 7). The slope for intact muscle, however, was not as well defined as that for skinned fibers due to the greater difficulty in controlling cytosolic Pi in living muscle. Only two Pi concentrations were achieved for intact mouse soleus (Figure 7A), and the leftmost point (low [Pi] in the presence of pyruvate) is an upper limit for [Pi] because the limit of detection by ^31^P-NMR is ~1 mM. Thus, the slope for intact muscle could be less steep. Regardless, the relationship between isometric force, [Pi], and ΔG_ATP_ applies whether mechanical events leading to force generation occur prior to or after release of Pi from the myosin head [65,88,89,94,95,96,97].

In contrast to Pi, the mechanism of force inhibition by pH is difficult to predict because protons can participate in the crossbridge cycle in multiple ways. During the ATPase cycle, proton release (stoichiometric coefficient α in Equation (2)) occurs simultaneously with Pi release because the affinity for H^+^ of the phosphate moiety changes when it is cleaved from the terminal γ position within the ATP molecule. Thus, it is reasonable to assume that isometric force should vary with [H^+^] in a manner analogous to that observed with [Pi]. While the data of Nosek, Fender and Godt [46] are consistent with this hypothesis, subsequent measurements have suggested that this is not the case [35,60,98]. The present study provides further evidence that the effects of pH are distinct from those of Pi (Figure 7 and Figure 8).

When interpreted in terms of ΔG_ATP_, the effects of pH on isometric force observed in living muscle vary in a consistent manner with observations in permeabilized muscle regardless of fiber type, i.e., all of the relationships in Figure 8A have a positive slope. In addition, the slopes for fast muscles in Figure 8A are steeper compared with those for slow muscles. Studies on the direct effects of altered pH on muscle force generation indicate that force inhibition by H^+^ is lower at physiological temperatures than at the lower temperatures that have often been used for experiments on reduced systems [53,98], which likely explains all or part of the differences in slopes between skinned fiber data obtained at cooler temperatures than data for intact muscle (Figure 8A). At 37 °C, acidification of isolated muscles did not affect the energetic cost of contraction [67]. The inhibition of isometric force by Pi (e.g., Figure A3A,B) is similarly reduced as temperature is increased close to physiological levels [45,48,62].

The positive slopes of the relationships between isometric force and ΔG_ATP_ when pH was varied (Figure 8A) are opposite to what was observed when [Pi] was varied (Figure 7). Significantly, the positive slopes in Figure 8A are opposite both to expectations and what is energetically possible [45,59,92]. Therefore, we conclude that it is not ΔG_ATP_ per se that determines isometric force production but the free energy associated with a specific step or steps associated with Pi release in the actomyosin ATPase cycle.

In search of an explanation of the effects of pH on force, we took advantage of the fact that our results for determining CK K_eq_″ over a wide range of conditions (Figure 6 and Equation (13)) allowed us to determine [ADP] for the pH solutions. The results (Figure 8B) appear consistent with high-affinity (nM) binding of MgADP to the myosin head. However, additional experiments are required to distinguish the effects of pH from those due to nucleotides.

## 4. Materials and Methods

### 4.1. Solution Composition for Biochemical Analyses

All chemicals and enzymes were of the highest degree of purity available and were obtained from Sigma Chemical Co. (St. Louis, MO, USA). Solutions for biochemical analyses were designed to mimic cytosolic composition over a broad range of physiological conditions and temperatures, and were constructed using known binding constants for each species (Table A2) [76,99]. Solution composition (in mM) was 145 Na^+^, 6.5 K^+^, 2.5 EGTA (pCa 9), 8 MgATP, 30 PCr, and 1 Pi. No ADP was added. pMg (−log [Mg^2+^], where [Mg^2+^] is in molar units) was either 2, 3, or 4, as estimates of intracellular [Mg^2+^]_i_ are typically within that range [30,100]. The pH range of 6–8 was examined because it is the most physiologically and experimentally relevant range [101,102,103]; pH buffer was 50 mM MES at pH 6 and 6.5, 50 mM MOPS at pH 7, or 50 mM TES at pH 7.5 and 8.0 to achieve optimal buffering based on the buffer pK_a_s. In all solutions for biochemical analyses we used Γ/2 = 0.25 M, with the ionic balance adjusted using acetate as the anion and Tris as the cation. The solutions were titrated to their final pH at each experimental temperature (10, 20, 30, or 40 °C); thus, there were 60 combinations of pH, pMg, and temperature, covering the range of physiologically relevant conditions as well as those most commonly used in biochemical experiments. To determine K_eq_, CK was added at ~75 units/mL. Under each of the 60 combinations, either no Cr was added or 0.5, 5, or 50 mM Cr was added immediately prior to addition of CK, leading to four discrete values of Cr. This allowed us to manipulate ATP/ADP at each condition (240 solutions in total).

Solution composition was calculated using a program that utilized the National Institute of Standards and Technology (NIST) Critically Selected Stability Constants of Metal Complexes Database [76,99,104]. The desired [H^+^] was calculated with correction for Γ/2 and temperature using the following equation from Khoo [105]:

(18) $${\left[H^{+}\right]}_{\mathrm{desired}}=10^{-pH+\frac{\sqrt{\frac{\Gamma }{2}}\left(0.5+0.000813\;T\right)}{\left(1+1.394\sqrt{\frac{\Gamma }{2}}\right)}-\sqrt{\frac{\Gamma }{2}}\left(0.08885-0.000111\;T\right)}$$

where T is the desired temperature (°C). The first protonation of Cr and the equivalent protonation of PCr were not included in calculations because K_a_ > 10^14^ for both, meaning that protonation is essentially complete over the entire 6–8 pH range.

### 4.2. Nuclear Magnetic Resonance Spectroscopy

Phosphorus NMR (^31^P-NMR) spectroscopy was performed on two high-field spectrometers. For equilibration studies, the spectrometer was a 7T GN 300 (Bruker Instruments, Billerica, MA, USA) using a 10 mm broadband commercial NMR probe tuned to the phosphorus frequency (121 MHz). A subset of experiments was performed at higher field strengths using a Varian 600 MHz Anova spectrometer (Varian, Palo Alto, CA) at the phosphorus frequency (242 MHz). A representative series of ^31^P-NMR spectra obtained at 600 MHz over the entire pH range 6–8, pMg 3.0, 30 °C, and 50 mM added Cr is shown in Figure 1. Magnetic field homogeneity was shimmed (usually less than 0.07 PPM) on the available proton signal prior to the start of the experiment. Data were acquired at 300 MHz with a π/2 pulse width (18 μs at 90 W), 15 s delay, and 4K data points. Transformed data were the sum of 64 acquisitions that were apodized with a 3 Hz exponential filter prior to Fourier transformation. All experiments were referenced to an external standard of dilute phosphoric acid (δ = 0 PPM) that was placed in a small glass capillary and positioned concentrically in the center of the NMR tube.

To confirm equilibration of the CK reaction in each solution, serially acquired ^31^P-NMR data from each solution were obtained at 300 MHz. The temperature was maintained (±1 °C) by a blanket of dry N_2_ gas that was first passed through a set of copper coils immersed in either a water bath or an acetone–dry ice bath, depending on the desired temperature. The solution temperature was measured using a thermocouple immersed in the solution while placed within the probe. Spectra were acquired while avoiding saturation of spectral resonances (recycle time 5*T1s), then an aliquot was removed for later analysis. At this point, the phosphorylation reaction was initiated by the addition of CK (~75 units/mL) to each tube and the tube was returned to the spectrometer for further acquisitions in order to follow the approach to equilibration. Serial spectra were acquired during the time course to equilibration and were halted when the change in peak area for PCr differed by less than 5% from the previous acquisition. At equilibration, the sample was removed from the magnet, kept at constant temperature by rapid immersion in a water bath, and CK was denatured by the addition of 2% SDS per mg of CK as previously described [32]. Samples were then frozen at −70 °C for later chemical analysis by anion and cation HPLC.

Analysis of spectral areas and chemical shift positions was performed on summed data processed with a 3 Hz line broadening and zero-filled one time prior to Fourier transformation. Spectra were analyzed for peak positions and integral areas using commercially available software (Bruker Instruments, Billerica, MA, USA). To obtain pH titration curves for Pi at each pMg and temperature (Equation (7)), the chemical shift of Pi (δ_Pi_) relative to the external standard was obtained from NMR spectra as adapted from Kost [55] with more comprehensive consideration of the ionic interactions.

### 4.3. HPLC Analysis

Chromatographic analysis was performed on stable SDS-treated samples as previously described using a Waters Millennium HPLC system (Waters Corp, Milford, MA, USA) [106]. In brief, nucleotides and PCr content were analyzed using a Vydac 303NT405 NTP anion exchange column (Vydac, Hesperia, CA, USA) with a phosphate gradient from 50 mM (pH 4.5) to 400 mM (pH 2.7) linearly applied over 20 min (Figure 3A,B). Creatine was determined by cation exchange chromatography using a Waters amino acid column under isocratic conditions with 25 mM sodium phosphate (pH 7.8) (Figure 3C,D). Detection of all analytes was by absorbance at 210 nm and quantification was from calibration curves determined using known standards.

### 4.4. Muscle Mechanics

In order to examine the relationship between muscle mechanical parameters and ΔG_ATP_, we utilized data from previously published studies on muscle mechanics of permeabilized fibers [35,36] and mechanics along with biochemical analyses of isolated muscle tissues [49,67]. All protocols for harvesting muscle tissue from animals were in accordance with the policies and standards of the National Institutes of Health/National Research Council Guide for the Care and Use of Laboratory Animals. Muscle tissues were obtained according to protocols approved by the Institutional Animal Care and Use Committee (IACUC) as described in the original publications and at Michigan State University and Florida State University (approved protocol 0118).

#### 4.4.1. Single Permeabilized Muscle Fiber Studies

Single chemically permeabilized (“skinned”) muscle fibers were dissected from rabbit psoas or soleus muscles and prepared for mechanical experimentation using published methods [35,36,53,104,107,108]. Single fiber segments (length ~ 2 mm) were isolated in a cold bath (4 °C) of 50% glycerol-relaxing solution and the fiber ends were chemically cross-linked by localized microapplication of chemical fixative (5% glutaraldehyde plus 1 mg/mL fluorescein for visualization) to generate ‘artificial tendons’ that minimize end compliance. The fixed ends were wrapped in Al foil ‘T clips’ (KEM-MIL, Hayward, CA, USA) and the T-clips were placed on hooks on a motor and force transducer (using silicone adhesive for stabilization) mounted on the modified stage of a Leitz Diavert (Wetzlar, Germany) inverted microscope for mechanical measurements.

Activating (pCa < 5) and relaxing (pCa > 8) solutions for fiber mechanics experiments were prepared as described in [35,36]. The composition of the control solution (in mM) was 5 MgATP, 1 Pi, 4 EGTA, 15 PCr, 100 monovalent cations (sum of K^+^ plus Na^+^), 3 Mg^2+^ (pMg 2.52), 50 MOPS, and 1 mg/mL CK. Control pH was 7.1 and was adjusted at 12 °C (the experimental temperature). When pH was varied, the pH buffer was varied as well to maintain buffering capacity, as follows: MES at pH 6.0 and 6.5; MOPS at pH 7.1 (control) and 7.3; MOPS, HEPES, or TES at pH 7.5; and EPPS at pH 8.0. [Ca^2+^] was adjusted by adding appropriate amounts of Ca(acetate)_2_; EDTA was substituted for EGTA when it was more appropriate as the Ca^2+^ buffer, taking into consideration Mg^2+^ binding by EDTA. [Pi] was varied from 0.1 mM (no added Pi) to 36 mM. Γ/2 = 0.16 M in all solutions for permeabilized fiber mechanics, with the ionic balance adjusted using Tris as the cation and acetate as the anion.

Experimental control, data acquisition, and data analysis were accomplished using custom software described previously [35,36,53,104,107,108]. The stability of the fiber structure and mechanical properties during activation were maintained by transient shortening of the fibers every 5 s at a rate that was at least as fast as the maximum shortening velocity, which reduced the force to zero; periodic unloading was followed by rapid re-stretching to the initial isometric length (L_0_). The initial sarcomere length (L_s_) was set to 2.6 μm in relaxing conditions. Following a brief initial control activation, fibers were returned to relaxing solution, L_s_ was adjusted if necessary, and L_0_, fiber diameter, and passive force were measured. Maximum isometric force was determined first in control conditions, then in an experimental condition (varied pH or [Pi]), then in control conditions. Normalized force was calculated as the experimental force divided by the average of the two bracketing controls. The similarity of force during first and last activations indicated that the fiber structure and function were stable under the examined conditions.

V_US_ was measured at maximum Ca^2+^-activation using the slack test [109] adapted as described previously in [35,36]. Normalized force and normalized V_US_ were calculated as the experimental value divided by the average of the two bracketing controls.

#### 4.4.2. Studies on Intact Muscles from Mice

Isolated mouse muscle experiments were conducted as described in [49,53,110,111], with modifications. Both soleus (SOL) muscles were ligated at the proximal and distal tendons with 5.0 silk sutures, removed from the hindlimbs, and immediately placed in organ baths. Muscles were incubated in modified mouse Ringer’s solution (in mM: 117 NaCl, 4.6 KCl, 25 NaHCO_3_, 2.5 CaCl_2_, 1.16 MgSO_4_, and 11 glucose) containing 10 mg/L gentamycin and equilibrated with 95% O_2_/5% CO_2_. The pH was 7.4 at 37 °C. Superfusate temperature was measured in a subset of experiments using a K-type thermocouple (Omega Engineering, Stamford, CT, USA) adjacent to the muscle and maintained at 37 ± 0.2 °C by circulating water through a glass-jacketed organ bath (Radnoti Glass Technology, Inc., Monrovia, CA, USA).

Isolated SOL muscles were mounted for mechanical measurements by tying one end of the muscle ligature to a stationary hook and the other end to an isometric force transducer fitted on a micrometer. Muscles were aligned with the axis of the transducer and the length was adjusted to optimal resting length (L_o_) using the length–tension relationship. Electrical stimulation was delivered via two Pt plate electrodes adjacent to the muscle and generated using a Grass S88 Stimulator (Grass Instruments, Quincy, MA, USA). The pulse duration was 0.2 ms and stimulation trains for tetanic force were delivered at fusion frequency (~70 Hz) for 0.5–1.2 s. Force was recorded using an ADC model AT MIO16E (National Instruments, Austin, TX, USA) controlled by commercially available software (LabScribeNI, iWorx, Dover, NH, USA). Analysis of mechanical transients was performed using a custom algorithm for physiological data developed in this laboratory using the MATLAB programming environment (MathWorks, Natick, MA, USA) [112].

### 4.5. Statistical Analysis

Nonlinear regression analyses were initially performed using SigmaPlot version 8.0 (SPSS Inc., Richmond, CA, USA) and validated using R version 4.0.5 or later. Regression parameter estimates are provided ± the standard error (SE) of the regression.

## Figures and Tables

**Table 1:** Regression coefficients for pH titrations of Pi from ^31^P-NMR experiments and calculated predictions at three different free magnesium concentrations. Values are nonlinear least squares regression parameter estimates ± SE for the curves shown in Figure 2. Rows labeled NMR are parameter estimates for ^31^P-NMR chemical shift data fitted to Equation (8) (Figure 2A–C). Rows labeled “calc” are parameter estimates for regression of Pi titration calculated predictions from binding equilibria as described in Section 4.1 fitted to Equation (9) (Figure 2D–F).

		pMg		
		2	3	4
pK_a_	NMR	6.66 ± 0.05	6.85 ± 0.05	6.85 ± 0.05
	calc	6.674 + 0.002	6.781 ± 0.002	6.796 ± 0.003
*d*pK_a_/*d*T	NMR	−0.013 ± 0.004	−0.016 ± 0.003	−0.015 ± 0.003
	calc	−0.0092 ± 0.0001	−0.0089 ± 0.0001	−0.0088 ± 0.0001
Δ	NMR	0.06 ± 0.02	0.25 ± 0.03	0.15 ± 0.03
*A*	calc	0.967 ± 0.002	0.955 ± 0.002	0.953 ± 0.002

## Data Availability

The data presented in this study are available in an Excel File S1 (subdivided with tabs) that is provided as a Supplement. The final tab in the Excel file allows the reader to estimate [ADP] and ΔG_ATP_ from their own data according to the algorithm described herein.

## Supplementary Materials

The following supporting information can be downloaded at: https://www.mdpi.com/article/10.3390/ijms241713244/s1.

## Appendix B

Section 2.2.1 discusses the proton stoichiometric coefficient β (Equation (4)) in the context of obtaining estimates of K_eq_″ [pMg, T] (Figure 4, Figure 5 and Figure 6). Here, we briefly examine estimation of the proton stoichiometric coefficient α (Equation (2)), even though it is not utilized elsewhere in this study.

Combining Equations (2) and (4) brings together the two proton stoichiometric coefficients α (Equation (2)) and β (Equation (4)) in an equation that describes hydrolysis of PCr, which is the net reaction that occurs during brief contractions of healthy living muscle:

PCr + (β − α)H^+^ → Cr + Pi (A1)

The metal ion binding affinities for PCr and Cr are outside of the physiological pH range (Section 4.1 and Table A2); thus, β – α can be estimated from the physiologically relevant protonation state of Pi over the range of conditions covered in this study (Figure 2 and Figure 4). The coefficient α can be estimated on its own from the ion binding equilibria (Section 4.1 and Table A2) comparably to calculations that were described to obtain coefficient β (Results Section 2.2.1).

## Appendix C

To ensure that the data in Figure 7 reflect the maximum Ca^2+^-activated isometric force, we measured force–pCa relations in permeabilized fibers from rabbit psoas (Figure A2A; N = 4 fibers) and soleus (Figure A2B; N = 3 fibers) at 0.1 mM Pi (control) and 20 mM Pi. Isometric force–pCa data were fitted to a version of the Hill Equation for cooperative binding [113]:

(A2) $${Force}_{normalized}={F_{max}\left(1+10^{\left(n_{Hill}\left(pCa-{pCa}_{50}\right)\right)}\right)}^{-1}$$

The dependent variable in Equation (A2) is the normalized isometric force (Force_normalized_), which was obtained by first subtracting the passive force (the small amount of force measured at pCa 8) from all force measurements from the same fiber at the same [Pi] and second by dividing the remaining active force by the active force at pCa 4.5 and 0.1 mM Pi for the same fiber. Thus, F_max_ was constrained to be 1.0 for the regression of data at 0.1 mM Pi, while it was a variable in the two regressions at 20 mM Pi. The parameter pCa_50_ in Equation (A2) corresponds to the pCa required to achieve F_max_/2, while n_Hill_ is related to the slope at pCa_50_ and is generally considered to reflect cooperativity in the process of Ca^2+^ activation of force. Table A1 contains the parameter estimates from nonlinear regression for the four fits to Equation (A2) shown in Figure A2. The rightward shift (ΔpCa_50_), corresponding to a decrease in Ca^2+^ sensitivity due to added Pi, was 0.11 pCa units for psoas and 0.13 pCa units for soleus (Table A1).

**Table A1:** Parameter estimates from nonlinear regressions of force–pCa data at 0.1 or 20 mM Pi from single permeabilized fibers from rabbit psoas (Figure A2A) and soleus (Figure A2B) muscles. The parameter estimates correspond to the four lines in Figure A2 when each of the four datasets was fitted to the Hill Equation (Equation (A2)). All values for pCa_50_ and n_Hill_ are provided ± SE regression, as are values for F_max_ at 20 mM Pi.

	[Pi] (mM)	F_max_	pCa_50_	n_Hill_
Rabbit psoas	0.1	1.0	5.90 ± 0.01	2.85 ± 0.18
	20.0	0.67 ± 0.01	5.79 ± 0.01	3.89 ± 0.52
Rabbit soleus	0.1	1.0	5.88 ± 0.02	1.95 ± 0.17
	20.0	0.77 ± 0.02	5.75 ± 0.03	2.14 ± 0.25

For comparison with prior studies, we plotted the maximum isometric force versus Pi data for permeabilized psoas (Figure A3A; N = 28 fibers) and soleus (Figure A3B; N = 19 fibers) muscle fibers. These data suggest that the maximum isometric force of soleus fibers is more affected by Pi at lower concentrations compared with psoas fibers, with an apparent binding affinity (K_m_) of 2.4 ± 0.7 mM versus 17.5 ± 5.2 mM, respectively, when the data were fitted to Equation (A3):

(A3) $${Force}_{normalized}\left(\left[Pi\right]\right)=1-\left(\frac{\left(1-F\left(\infty \right)\right)\left(\left[Pi\right]-0.1\right)}{K_{m}+\left(\left[Pi\right]-0.1\right)}\right)$$

The dependent variable in Equation (A3) is the normalized isometric force (Force_normalized_([Pi])), which was obtained by first subtracting the passive force (the small amount of force measured at pCa 8) from all force measurements from the same fiber and second by dividing the remaining active force by the active force at saturating Ca^2+^ and 0.1 mM Pi for the same fiber. Thus, the normalized force was 1.0 for the data point at 0.1 mM Pi. Note that Equation (A3), which includes an asymptotic force to which the isometric force declines at very high levels of Pi (F(∞)), represents a model that is not entirely consistent with the alternate model implied in Figure 7.

Combined with the decrease in Ca^2+^ sensitivity with increased Pi (Figure A2), the decrease in maximal isometric force in both fast and slow fiber types (the right side of each panel in Figure A2 and Figure A3A,B) indicates that substantially more Ca^2+^ would be required to achieve the same force levels in the presence of elevated Pi, while the loss of force due to the rightward shifts of Ca^2+^ sensitivity (Figure A2) could potentially be overcome only at the low end of the submaximal range of Ca^2+^ concentrations due to the loss of maximal isometric force (Figure A3A,B).

To test for nonspecific effects of multivalent anions, we examined the effects of sulfate ([SO_4_^=^]) on the maximum isometric force of permeabilized fibers from rabbit psoas (Figure A3C; N = 7 fibers) and soleus (Figure A3D; N = 7 fibers) muscles. All measurements were made in the presence of 0.1 mM Pi, and no added sulfate was the control condition used for normalizing force, as described above. Comparing the small effects of sulfate (Figure A3C,D) with the effects of Pi (Figure A3A,B) on the isometric force of permeabilized muscle fibers suggests that the effects of Pi are specific for that anion.

In addition, we examined the effects of [Pi] on the velocity of unloaded shortening (V_US_) as measured using the slack test [109] (Section 4.4.1). The data in Figure A4 indicate that there is a small inhibitory effect of Pi in both fiber types, with the effect being slightly larger in soleus fibers (Figure A4B). The effect of Pi on V_US_ (Figure A4A) is clearly smaller than its effect on the isometric force (Figure A3A), consistent with the expectation that ΔG_ATP_ is not limiting for unloaded shortening.

## Appendix D

**Table A2:** Summary of the binding constants used to construct model solutions and calculations of proton stoichiometric coefficients, showing the metal ion-binding constants used for calculating the solutions (Section 4.1) and H^+^ stoichiometric constants α (Equation (2)) and β (Equation (4) and Figure 4).

Equilibrium	log K_eq_	log K_eq_	log K_eq_	log K_eq_	Reference(s)
	10 °C	20 °C	30 °C	40 °C	
[HATP^3−^]/[H^+^][ATP^4−^]	6.71	6.72	6.72	6.73	[114,115]
[H_2_ATP^2−^]/[H^+^][HATP^3−^]	3.87	3.88	3.89	3.90	[114,115]
[H_3_ATP^−^]/[H^+^][H_2_ATP^2−^]	1.80	1.80	1.80	1.80	[115]
[MgATP^2−^]/[Mg^2+^][ATP^4−^]	4.37	4.38	4.39	4.40	[114,115]
[MgHATP^−^]/[Mg^2+^][HATP^3−^]	2.11	2.13	2.14	2.15	[114,115]
[Mg_2_ATP]/[Mg^2+^][MgATP^2−^]	1.70	1.70	1.70	1.70	[115]
[CaATP^2−^]/[Ca^2+^][ATP^4−^]	4.06	4.07	4.08	4.09	[114,115]
[CaHATP^−^]/[Ca^2+^][HATP^3−^]	1.95	1.97	1.98	1.99	[114,115]
[NaATP^3−^]/[Na^+^][ATP^4−^]	1.23	1.24	1.24	1.25	[114,115]
[KATP^3−^]/[K^+^][ATP^4−^]	1.09	1.10	1.10	1.11	[114,115]
[HADP^2−^]/[H^+^][ADP^3−^]	6.47	6.48	6.48	6.49	[114,115]
[H_2_ADP^−^]/[H^+^][HADP^2−^]	3.82	3.83	3.84	3.85	[114,115]
[MgADP^−^]/[Mg^2+^][ADP^3−^]	3.25	3.26	3.27	3.28	[114,115]
[MgHADP]/[Mg^2+^][HADP^2−^]	1.40	1.42	1.43	1.44	[114,115]
[CaADP^−^]/[Ca^2+^][ADP^3−^]	2.90	2.91	2.92	2.93	[114,115]
[CaHADP]/[Ca^2+^][HADP^2−^]	1.44	1.46	1.47	1.48	[114,115]
[NaADP^2−^]/[Na^+^][ADP^3−^]	1.04	1.05	1.05	1.06	[114,115]
[KADP^2−^]/[K^+^][ADP^3−^]	0.92	0.93	0.93	0.94	[114,115]
[HPO_4_^2−^]/[H^+^][PO_4_^3−^]	12.24	12.15	12.07	11.98	[86,115]
[H_2_PO_4_^−^]/[H^+^][HPO_4_^2−^]	6.96	6.94	6.92	6.90	[86,115]
[H_3_PO_4_]/[H^+^][H_2_PO_4_^−^]	1.81	1.86	1.90	1.95	[86,115]
[MgHPO_4_]/[Mg^2+^][HPO_4_^2−^]	2.25	2.33	2.40	2.48	[86,115,116]
[CaHPO_4_]/[Ca^2+^][HPO_4_^2−^]	2.11	2.19	2.26	2.34	[86,115,116]
[NaPO_4_^2−^]/[Na^+^][PO_4_^3−^]	0.75	0.75	0.75	0.75	[115]
[NaHPO_4_^−^]/[Na^+^][HPO_4_^2−^]	0.49	0.49	0.49	0.49	[115]
[KPO_4_^2−^]/[K^+^][PO_4_^3−^]	0.60	0.60	0.60	0.60	[115]
[KHPO_4_^−^]/[K^+^][HPO_4_^2−^]	0.35	0.35	0.35	0.35	[115]
[HCP^−^]/[H^+^][CP^2−^]	4.58	4.58	4.58	4.58	[117,118,119]
[H_2_CP]/[H^+^][HCP^−^]	2.70	2.70	2.70	2.70	[117,118,119]
[H_3_CP^+^]/[H^+^][H_2_CP]	2.00	2.00	2.00	2.00	[117,118,119]
[MgCP]/[Mg^2+^][CP^2−^]	1.60	1.60	1.60	1.60	[116,117]
[CaCP]/[Ca^2+^][CP^2−^]	1.30	1.30	1.30	1.30	[116,117]
[NaCP^−^]/[Na^+^][CP^2−^]	0.74	0.74	0.74	0.74	[117]
[NaHCP]/[Na^+^][HCP^−^]	0.31	0.31	0.31	0.31	[117]
[KCP^−^]/[K^+^][CP^2−^]	0.74	0.74	0.74	0.74	[117]
[KHCP]/[K^+^][HCP^−^]	0.31	0.31	0.31	0.31	[117]
[HCr^+^]/[H^+^][Cr]	2.63	2.63	2.63	2.63	[117]
[HEGTA^3−^]/[H^+^][EGTA^4−^]	9.39	9.25	9.11	8.96	[118,120]
[H_2_EGTA^2−^]/[H^+^][HEGTA^3−^]	8.83	8.69	8.55	8.41	[118,120]
[H_3_EGTA^−^]/[H^+^][H_2_EGTA^2−^]	2.57	2.57	2.57	2.57	[118,120]
[H_4_EGTA]/[H^+^][H_3_EGTA^−^]	1.97	1.97	1.97	1.97	[118,120]
[MgEGTA^2−^]/[Mg^2+^][EGTA^4−^]	4.65	4.77	4.90	5.02	[118,120]
[MgHEGTA^−^]/[Mg^2+^][HEGTA^3−^]	2.98	2.98	2.98	2.98	[118,120]
[CaEGTA^2−^]/[Ca^2+^][EGTA^4−^]	10.65	10.45	10.26	10.06	[115,118,119,120,121]
[CaHEGTA^−^]/[Ca^2+^][HEGTA^3−^]	4.91	4.91	4.91	4.91	[115,118,119,120,121]
[NaEGTA^3−^]/[Na^+^][EGTA^4−^]	1.66	1.66	1.66	1.66	[120]
[KEGTA^3−^]/[K^+^][EGTA^4−^]	0.96	0.96	0.96	0.96	[120]
[HMES]/[H^+^][MES^−^]	6.29	6.18	6.07	5.96	[115,122]
[HMOPS]/[H^+^][MOPS^−^]	7.28	7.13	6.98	6.83	[115,122]
[HTES]/[H^+^][TES^−^]	7.71	7.51	7.31	7.11	[115,122]
[HTris^+^]/[H^+^][Tris]	8.68	8.37	8.06	7.75	[115,122]
[HOAc]/[H^+^][OAc^−^]	4.56	4.56	4.56	4.56	[115]
[MgOAc^+^]/[Mg^2+^][OAc^−^]	0.55	0.55	0.55	0.55	[115]
[CaOAc^+^]/[Ca^2+^][OAc^−^]	0.57	0.57	0.57	0.57	[115]
[NaOAc]/[Na^+^][OAc^−^]	−0.26	−0.26	−0.26	−0.26	[115]
[KOAc]/[K^+^][OAc^−^]	−0.41	−0.41	−0.41	−0.41	[115]

## References

[1] Kushmerick M.J., Peachey L.D. (1983). Energetics of muscle contraction. Handbook of Physiology—Skeletal Muscle.

[2] Dumas J.F., Simard G., Flamment M., Ducluzeau P.H., Ritz P. (2009). Is skeletal muscle mitochondrial dysfunction a cause or an indirect consequence of insulin resistance in humans?. Diabetes Metab..

[3] Petersen K.F., Shulman G.I. (2006). New insights into the pathogenesis of insulin resistance in humans using magnetic resonance spectroscopy. Obesity (Silver Spring).

[4] Wallis R.H., Collins S.C., Kaisaki P.J., Argoud K., Wilder S.P., Wallace K.J., Ria M., Ktorza A., Rorsman P., Bihoreau M.T. (2008). Pathophysiological, genetic and gene expression features of a novel rodent model of the cardio-metabolic syndrome. PLoS ONE.

[5] Befroy D.E., Petersen K.F., Dufour S., Mason G.F., de Graaf R.A., Rothman D.L., Shulman G.I. (2007). Impaired mitochondrial substrate oxidation in muscle of insulin-resistant offspring of type 2 diabetic patients. Diabetes.

[6] Johnson G., Roussel D., Dumas J.F., Douay O., Malthiery Y., Simard G., Ritz P. (2006). Influence of intensity of food restriction on skeletal muscle mitochondrial energy metabolism in rats. Am. J. Physiol. Endocrinol. Metab..

[7] Petersen K.F., Befroy D., Dufour S., Dziura J., Ariyan C., Rothman D.L., DiPietro L., Cline G.W., Shulman G.I. (2003). Mitochondrial dysfunction in the elderly: Possible role in insulin resistance. Science.

[8] Oltmanns K.M., Melchert U.H., Scholand-Engler H.G., Howitz M.C., Schultes B., Schweiger U., Hohagen F., Born J., Peters A., Pellerin L. (2008). Differential energetic response of brain vs. skeletal muscle upon glycemic variations in healthy humans. Am. J. Physiol. Regul. Integr. Comp. Physiol..

[9] Mathur M.C., Chase P.B., Chalovich J.M. (2011). Several cardiomyopathy causing mutations on tropomyosin either destabilize the active state of actomyosin or alter the binding properties of tropomyosin. Biochem. Biophys. Res. Commun..

[10] Muoio D.M., Newgard C.B. (2006). Obesity-related derangements in metabolic regulation. Annu. Rev. Biochem..

[11] Mandavia C.H., Aroor A.R., Demarco V.G., Sowers J.R. (2012). Molecular and metabolic mechanisms of cardiac dysfunction in diabetes. Life Sci..

[12] Catania C., Binder E., Cota D. (2011). mTORC1 signaling in energy balance and metabolic disease. Int. J. Obes. (Lond).

[13] Mudd J.O., Kass D.A. (2008). Tackling heart failure in the twenty-first century. Nature.

[14] Gafurov B., Fredricksen S., Cai A., Brenner B., Chase P.B., Chalovich J.M. (2004). The D14 mutation of human cardiac troponin T enhances ATPase activity and alters the cooperative binding of S1-ADP to regulated actin. Biochemistry.

[15] Bai F., Weis A., Takeda A.K., Chase P.B., Kawai M. (2011). Enhanced active cross-bridges during diastole: Molecular pathogenesis of tropomyosin’s HCM mutations. Biophys. J..

[16] Köhler J., Chen Y., Brenner B., Gordon A.M., Kraft T., Martyn D.A., Regnier M., Rivera A.J., Wang C.K., Chase P.B. (2003). Familial hypertrophic cardiomyopathy mutations in troponin I (K183D, G203S, K206Q) enhance filament sliding. Physiol. Genom..

[17] Kataoka A., Hemmer C., Chase P.B. (2007). Computational simulation of hypertrophic cardiomyopathy mutations in troponin I: Influence of increased myofilament calcium sensitivity on isometric force, ATPase and [Ca^2+^]_i_. J. Biomech..

[18] Loong C.K.P., Zhou H.-X., Chase P.B. (2012). Familial hypertrophic cardiomyopathy related E180G mutation increases flexibility of human cardiac a-tropomyosin. FEBS Lett..

[19] Loong C.K.P., Badr M.A., Chase P.B. (2012). Tropomyosin flexural rigidity and single Ca^2+^ regulatory unit dynamics: Implications for cooperative regulation of cardiac muscle contraction and cardiomyocyte hypertrophy. Front. Physiol..

[20] Gonzalez-Martinez D., Johnston J.R., Landim-Vieira M., Ma W., Antipova O., Awan O., Irving T.C., Chase P.B., Pinto J.R. (2018). Structural and functional impact of troponin C-mediated Ca^2+^ sensitization on myofilament lattice spacing and cross-bridge mechanics in mouse cardiac muscle. J. Mol. Cell Cardiol..

[21] Montessuit C., Lerch R. (2013). Regulation and dysregulation of glucose transport in cardiomyocytes. Biochim. Biophys. Acta.

[22] Landim-Vieira M., Ma W., Song T., Rastegarpouyani H., Gong H., Leite Coscarella I., Bogaards S.J.P., Conijn S.P., Ottenheijm C.A.C., Hwang H.S. (2023). Cardiac troponin T N-domain variant destabilizes the actin interface resulting in disturbed myofilament function. Proc. Natl. Acad. Sci. USA.

[23] Phillips R.C., George P., Rutman R.J. (1969). Thermodynamic data for the hydrolysis of adenosine triphosphate as a function of pH, Mg^2+^ ion concentration, and ionic strength. J. Biol. Chem..

[24] George P., Rutman R.J. (1960). The “high energy phosphate bond” concept. Prog. Biophys. Mol. Biol..

[25] Alberty R.A. (1969). Standard Gibbs free energy, enthalpy, and entropy changes as a function of pH and pMg for several reactions involving adenosine phosphates. J. Biol. Chem..

[26] Alberty R.A. (1992). Equilibrium calculations on systems of biochemical reactions at specified pH and pMg. Biophys. Chem..

[27] Alberty R.A., Cornish-Bowden A. (1993). The pH dependence of the apparent equilibrium constant, K’, of a biochemical reaction. Trends Biochem. Sci..

[28] Schief W.R., Howard J. (2001). Conformational changes during kinesin motility. Curr. Opin. Cell Biol..

[29] Lawson J.W.R., Veech R.L. (1979). Effects of pH and free Mg^2+^ on the K_eq_ of the creatine kinase reaction and other phosphate hydrolyses and phosphate transfer reactions. J. Biol. Chem..

[30] Kushmerick M.J., Dillon P.F., Meyer R.A., Brown T.R., Krisanda J.M., Sweeney H.L. (1986). ^31^P NMR spectroscopy, chemical analysis, and free Mg^2+^ of rabbit bladder and uterine smooth muscle. J. Biol. Chem..

[31] Kushmerick M.J., Moerland T.S., Wiseman R.W. (1993). Two classes of mammalian skeletal muscle fibers distinguished by metabolite content. Adv. Exp. Med. Biol..

[32] Wiseman R.W., Kushmerick M.J. (1995). Creatine kinase equilibration follows solution thermodynamics in skeletal muscle. 31P NMR studies using creatine analogs. J. Biol. Chem..

[33] Wiseman R.W., Kushmerick M.J. (1997). Phosphorus metabolite distribution in skeletal muscle: Quantitative bioenergetics using creatine analogs. Mol. Cell Biochem..

[34] Brault J.J., Abraham K.A., Terjung R.L. (2003). Phosphocreatine content of freeze-clamped muscle: Influence of creatine kinase inhibition. J. Appl. Physiol. (1985).

[35] Chase P.B., Kushmerick M.J. (1988). Effects of pH on contraction of rabbit fast and slow skeletal muscle fibers. Biophys. J..

[36] Chase P.B., Kushmerick M.J. (1995). Effect of physiological ADP concentrations on contraction of single skinned fibers from rabbit fast and slow muscles. Am. J. Physiol..

[37] Meyer R.A., Kushmerick M.J., Brown T.R. (1982). Application of ^31^P-NMR spectroscopy to the study of striated muscle metabolism. Am. J. Physiol..

[38] Eisenberg E., Hill T.L. (1978). A cross-bridge model of muscle contraction. Prog. Biophys. Mol. Biol..

[39] Eisenberg E., Hill T.L. (1985). Muscle contraction and free energy transduction in biological systems. Science.

[40] Howard J. (2001). Mechanics of Motor Proteins and the Cytoskeleton.

[41] Pate E., Cooke R. (1989). A model of crossbridge action: The effects of ATP, ADP, and Pi. J. Muscle Res. Cell Motil..

[42] Jeneson J.A.L., Westerhoff H.V., Brown T.R., Van Echteld C.J.A., Berger R. (1995). Quasi-linear relationship between Gibbs free energy of ATP hydrolysis and power output in human forearm muscle. Am. J. Physiol..

[43] Westerhoff H.V., van Echteld C.J.A., Jeneson J.A.L. (1995). On the expected relationship between Gibbs energy of ATP hydrolysis and muscle performance. Biophys. Chem..

[44] Godt R.E., Nosek T.M. (1989). Changes in intracellular milieu with fatigue or hypoxia depress contraction of skinned rabbit skeletal and cardiac muscle. J. Physiol..

[45] Karatzaferi C., Chinn M.K., Cooke R. (2004). The force exerted by a muscle cross-bridge depends directly on the strength of the actomyosin bond. Biophys. J..

[46] Nosek T.M., Fender K.Y., Godt R.E. (1987). It is diprotonated inorganic phosphate that depresses force in skinned skeletal muscle fibers. Science.

[47] Cooke R., Franks K., Luciani G.B., Pate E. (1988). The inhibition of rabbit skeletal muscle contraction by hydrogen ions and phosphate. J. Physiol..

[48] Debold E.P., Dave H., Fitts R.H. (2004). Fiber type and temperature dependence of inorganic phosphate: Implications for fatigue. Am. J. Physiol. Cell Physiol..

[49] Phillips S.K., Wiseman R.W., Woledge R.C., Kushmerick M.J. (1993). The effect of metabolic fuel on force production and resting inorganic phosphate levels in mouse skeletal muscle. J. Physiol..

[50] Pate E., Lin M., Franks-Skiba K., Cooke R. (1992). Contraction of glycerinated rabbit slow-twitch muscle fibers as a function of MgATP concentration. Am. J. Physiol. Cell Physiol..

[51] Cooke R., Bialek W. (1979). Contraction of glycerinated muscle fibers as a function of the ATP concentration. Biophys. J..

[52] Cooke R., Pate E. (1985). The effects of ADP and phosphate on the contraction of muscle fibers. Biophys. J..

[53] Wiseman R.W., Beck T.W., Chase P.B. (1996). Effect of intracellular pH on force development depends on temperature in intact skeletal muscle from mouse. Am. J. Physiol..

[54] Vinnakota K.C., Beard D.A., Dash R.K. (2009). Design of experiments for identification of complex biochemical systems with applications to mitochondrial bioenergetics. Conf. Proc. IEEE Eng. Med. Biol. Soc..

[55] Kost G.J. (1990). pH standardization for phosphorus-31 magnetic resonance heart spectroscopy at different temperatures. Magn. Reson. Med..

[56] Adams G.R., Foley J.M., Meyer R.A. (1990). Muscle buffer capacity estimated from pH changes during rest-to-work transitions. J. Appl. Physiol..

[57] Harkema S.J., Meyer R.A. (1997). Effect of acidosis on control of respiration in skeletal muscle. Am. J. Physiol..

[58] Kushmerick M.J., Moerland T.S., Wiseman R.W. (1992). Mammalian skeletal muscle fibers distinguished by contents of phosphocreatine, ATP, and Pi. Proc. Natl. Acad. Sci. USA.

[59] Pate E., Franks-Skiba K., Cooke R. (1998). Depletion of phosphate in active muscle fibers probes actomyosin states within the powerstroke. Biophys. J..

[60] Nosek T.M., Leal-Cardoso J.H., McLaughlin M., Godt R.E. (1990). Inhibitory influence of phosphate and arsenate on contraction of skinned skeletal and cardiac muscle. Am. J. Physiol..

[61] Brune M., Hunter J.L., Corrie J.E.T., Webb M.R. (1994). Direct, real-time measurement of rapid inorganic phosphate release using a novel fluorescent probe and its application to actomyosin subfragment 1 ATPase. Biochemistry.

[62] Debold E.P., Romatowski J.G., Fitts R.H. (2006). The depressive effect of Pi on the force-calcium relationship in skinned single muscle fibers is temperature dependent. Am. J. Physiol. Cell Physiol..

[63] Fryer M.W., Owen V.J., Lamb G.D., Stephenson D.G. (1995). Effects of creatine phosphate and Pi on Ca^2+^ movements and tension development in rat skinned skeletal muscle fibres. J. Physiol..

[64] Martyn D.A., Gordon A.M. (1992). Force and stiffness in glycerinated rabbit psoas fibers. Effects of calcium and elevated phosphate. J. Gen. Physiol..

[65] Millar N.C., Homsher E. (1990). The effect of phosphate and calcium on force generation in glycerinated rabbit skeletal muscle fibers. J. Biol. Chem..

[66] Palmer S., Kentish J.C. (1994). The role of troponin C in modulating the Ca^2+^ sensitivity of mammalian skinned cardiac and skeletal muscle fibres. J. Physiol..

[67] Harkema S.J., Adams G.R., Meyer R.A. (1997). Acidosis has no effect on the ATP cost of contraction in cat fast- and slow-twitch skeletal muscles. Am. J. Physiol..

[68] Golding E.M., Golding R.M. (1995). Interpretation of ^31^P MRS spectra in determining intracellular free magnesium and potassium ion concentrations. Magn. Reson. Med..

[69] Iotti S., Frassineti C., Alderighi L., Sabatini A., Vacca A., Barbiroli B. (1996). In vivo assessment of free magnesium concentration in human brain by ^31^P MRS. A new calibration curve based on a mathematical algorithm. NMR Biomed..

[70] Headrick J.P., Willis R.J. (1989). Effect of inotropic stimulation on cytosolic Mg^2+^ in isolated rat heart: A ^31^P magnetic resonance study. Magn. Reson. Med..

[71] Teague W.E., Dobson G.P. (1992). Effect of temperature on the creatine kinase equilibrium. J. Biol. Chem..

[72] Teague W.E., Golding E.M., Dobson G.P. (1996). Adjustment of K’ for the creatine kinase, adenylate kinase and ATP hydrolysis equilibria to varying temperature and ionic strength. J. Exp. Biol..

[73] Golding E.M., Teague W.E., Dobson G.P. (1995). Adjustment of K’ to varying pH and pMg for the creatine kinase, adenylate kinase and ATP hydrolysis equilibria permitting quantitative bioenergetic assessment. J. Exp. Biol..

[74] Kushmerick M.J. (1997). Multiple equilibria of cations with metabolites in muscle bioenergetics. Am. J. Physiol..

[75] Fabiato A. (1988). Computer programs for calculating total from specified free or free from specified total ionic concentrations in aqueous solutions containing multiple metals and ligands. Methods Enzymol..

[76] Regnier M., Rivera A.J., Wang C.K., Bates M.A., Chase P.B., Gordon A.M. (2002). Thin filament near-neighbour regulatory unit interactions affect rabbit skeletal muscle steady-state force-Ca^2+^ relations. J. Physiol..

[77] Dweck D., Reyes-Alfonso Jr A., Potter J.D. (2005). Expanding the range of free calcium regulation in biological solutions. Anal. Biochem..

[78] Hardin C.D., Wiseman R.W., Kushmerick M.J. (1992). Vascular oxidative metabolism under different metabolic conditions. Biochim. Biophys. Acta.

[79] Kunzelmann S., Webb M.R. (2010). A fluorescent, reagentless biosensor for ADP based on tetramethylrhodamine-labeled ParM. ACS Chem. Biol..

[80] Botman D., van Heerden J.H., Teusink B. (2020). An Improved ATP FRET Sensor For Yeast Shows Heterogeneity During Nutrient Transitions. ACS Sens..

[81] Alberty R.A. (1968). Effect of pH and metal ion concentration on the equilibrium hydrolysis of adenosine triphosphate to adenosine diphosphate. J. Biol. Chem..

[82] Alberty R.A., San Pietro A., Gest H. (1972). Calculation of the standard Gibbs free energy, enthalpy, and entropy changes for the hydrolysis of ATP at 0 °C, 25 °C, 37 °C, and 75 °C. Horizons of Bioenergetics.

[83] Alberty R.A. (1998). Change in the binding of hydrogen ions and magnesium ions in the hydrolysis of ATP. Biophys. Chem..

[84] Alberty R.A., Goldberg R.N. (1992). Standard thermodynamic formation properties for the adenosine 5’-triphosphate series. Biochemistry.

[85] Rosing J., Slater E.C. (1972). The value of ΔG° for the hydrolysis of ATP. Biochim. Biophys. Acta.

[86] Phillips R.C., George P., Rutman R.J. (1963). Potentiometric studies of the secondary phosphate ionizations of AMP, ADP, and ATP, and calculations of thermodynamic data for the hydrolysis reactions. Biochemistry.

[87] Cooke R. (1997). Actomyosin interaction in striated muscle. Physiol. Rev..

[88] Millar N.C., Homsher E. (1992). Kinetics of force generation and phosphate release in skinned rabbit soleus muscle fibers. Am. J. Physiol. Cell Physiol..

[89] Dantzig J.A., Goldman Y.E., Millar N.C., Lacktis J., Homsher E. (1992). Reversal of the cross-bridge force-generating transition by photogeneration of phosphate in rabbit psoas muscle fibres. J. Physiol..

[90] Tesi C., Colomo F., Nencini S., Piroddi N., Poggesi C. (2000). The effect of inorganic phosphate on force generation in single myofibrils from rabbit skeletal muscle. Biophys. J..

[91] Pate E., Cooke R. (1989). Addition of phosphate to active muscle fibers probes actomyosin states within the powerstroke. Pflügers Arch..

[92] Pate E., Cooke R. (1988). Energetics of the actomyosin bond in the filament array of muscle fibers. Biophys. J..

[93] Tesi C., Colomo F., Piroddi N., Poggesi C. (2002). Characterization of the cross-bridge force-generating step using inorganic phosphate and BDM in myofibrils from rabbit skeletal muscles. J. Physiol..

[94] Takagi Y., Shuman H., Goldman Y.E. (2004). Coupling between phosphate release and force generation in muscle actomyosin. Philos. Trans. R. Soc. Lond. B Biol. Sci..

[95] Caremani M., Dantzig J., Goldman Y.E., Lombardi V., Linari M. (2008). Effect of inorganic phosphate on the force and number of myosin cross-bridges during the isometric contraction of permeabilized muscle fibers from rabbit psoas. Biophys. J..

[96] Muretta J.M., Rohde J.A., Johnsrud D.O., Cornea S., Thomas D.D. (2015). Direct real-time detection of the structural and biochemical events in the myosin power stroke. Proc. Natl. Acad. Sci. USA.

[97] Woody M.S., Winkelmann D.A., Capitanio M., Ostap E.M., Goldman Y.E. (2019). Single molecule mechanics resolves the earliest events in force generation by cardiac myosin. eLife.

[98] Pate E., Bhimani M., Franks-Skiba K., Cooke R. (1995). Reduced effect of pH on skinned rabbit psoas muscle mechanics at high temperatures: Implications for fatigue. J. Physiol..

[99] Liang B., Chen Y., Wang C.K., Luo Z., Regnier M., Gordon A.M., Chase P.B. (2003). Ca^2+^ regulation of rabbit skeletal muscle thin filament sliding: Role of cross-bridge number. Biophys. J..

[100] Csernoch L., Bernengo J.C., Szentesi P., Jacquemond V. (1998). Measurements of intracellular Mg^2+^ concentration in mouse skeletal muscle fibers with the fluorescent indicator mag-indo-1. Biophys. J..

[101] Meyer R.A., Brown T.R., Krilowicz B.L., Kushmerick M.J. (1986). Phosphagen and intracellular pH changes during contraction of creatine-depleted rat muscle. Am. J. Physiol..

[102] Margossian S.S., Lowey S. (1982). Preparation of myosin and its subfragments from rabbit skeletal muscle. Methods Enzymol..

[103] White H.D. (1982). Special instrumentation and techniques for kinetic studies of contractile systems. Meth Enzymol..

[104] Butcher M.T., Chase P.B., Hermanson J.W., Clark A.N., Brunet N.M., Bertram J.E. (2010). Contractile properties of muscle fibers from the deep and superficial digital flexors of horses. Am. J. Physiol. Regul. Integr. Comp. Physiol..

[105] Khoo R.H., Ramette R.W., Culberson C.H., Bates R.G. (1977). Determination of hydrogen ion concentrations in sea water from 5 to 40 °C: Standard potentials at salinities from 20 to 45%. Anal. Chem..

[106] Wiseman R.W., Moerland T.S., Chase P.B., Stuppard R., Kushmerick M.J. (1992). High-performance liquid chromatographic assays for free and phosphorylated derivatives of the creatine analogues b-guanidopropionic acid and 1-carboxy-methyl-2-iminoimidazolidine (cyclocreatine). Anal. Biochem..

[107] Schoffstall B., Clark A., Chase P.B. (2006). Positive inotropic effects of low dATP/ATP ratios on mechanics and kinetics of porcine cardiac muscle. Biophys. J..

[108] Schoffstall B., Kataoka A., Clark A., Chase P.B. (2005). Effects of rapamycin on cardiac and skeletal muscle contraction and crossbridge cycling. J. Pharmacol. Exp. Ther..

[109] Edman K.A.P. (1979). The velocity of unloaded shortening and its relation to sarcomere length and isometric force in vertebrate muscle fibres. J. Physiol..

[110] Phillips S.K., Wiseman R.W., Woledge R.C., Kushmerick M.J. (1993). Neither changes in phosphorus metabolite levels nor myosin isoforms can explain the weakness in aged mouse muscle. J. Physiol..

[111] Dentel J.N., Blanchard S.G., Ankrapp D.P., McCabe L.R., Wiseman R.W. (2005). Inhibition of cross-bridge formation has no effect on contraction-associated phosphorylation of p38 MAPK in mouse skeletal muscle. Am. J. Physiol. Cell Physiol..

[112] Jayaraman R.C., Latourette M.T., Siebert J.E., Wiseman R.W. (2006). A rapid algorithm for processing digital physiologic signals: Application to skeletal muscle contractions. Biomed. Signal Process Control..

[113] Hill A.V. (1910). The possible effects of the aggregation of the molecules of haemoglobin on its dissociation curves. J. Physiol..

[114] De Robertis A., De Stefano C., Sammartano S., Calì R., Purrello R., Rigano C. (1986). Alkali-metal and alkaline-earth-metal ion complexes with adenosine 5’-triphospate in aqueous solution. Thermodynamic parameters and their dependence on temperature and ionic strength. J. Chem. Res..

[115] Smith R.M., Martell A.E., Motekaitis R.J. (1995). NIST Critically Selected Stability Constants of Metal Complexes Database.

[116] O’Sullivan W.J., Smithers G.W. (1979). Stability constants for biologically important metal-ligand complexes. Methods Enzymol..

[117] Dawson R.M.C., Elliott D.C., Elliott W.H., Jones K.M. (1974). Data for Biochemical Research.

[118] Martell A.E., Smith R.M. (1974). Critical Stability Constants, Vol. 1: Amino Acids.

[119] Martell A.E., Smith R.M. (1982). Critical Stability Constants, Vol. 5: First Supplement.

[120] Harrison S.M., Bers D.M. (1989). Correction of proton and Ca association constants of EGTA for temperature and ionic strength. Am. J. Physiol. Cell Physiol..

[121] Robinson R.A., Stokes R.H. (1965). Electrolyte Solutions.

[122] Ellis K.J., Morrison J.F. (1982). Buffers of constant ionic strength for studying pH dependent processes. Methods Enzymol..

